# Recurrent Targeted Genes of Hepatitis B Virus in the Liver Cancer Genomes Identified by a Next-Generation Sequencing–Based Approach

**DOI:** 10.1371/journal.pgen.1003065

**Published:** 2012-12-06

**Authors:** Dong Ding, Xiaoyan Lou, Dasong Hua, Wei Yu, Lisha Li, Jun Wang, Feng Gao, Na Zhao, Guoping Ren, Lanjuan Li, Biaoyang Lin

**Affiliations:** 1 Hangzhou Proprium Biotech, Hangzhou, China; 2 Systems Biology Division and Proprium Research Center, Zhejiang–California International Nanosystems Institute (ZCNI), Zhejiang University, Hangzhou, China; 3 Department of General Surgery, The Second Affiliated Hospital, Shanxi Medical University, Taiyuan, China; 4 The First Affiliated Hospital, Zhejiang University, Hangzhou, China; 5 State Key Laboratory for Diagnosis and Treatment of Infectious Diseases, College of Medicine, Zhejiang University, Hangzhou, China; 6 Department of Urology, University of Washington, Seattle, Washington, United States of America; 7 Swedish Medical Center, Seattle, Washington, United States of America; Centre for Cancer Biology, SA Pathology, Australia

## Abstract

Integration of the viral DNA into host chromosomes was found in most of the hepatitis B virus (HBV)–related hepatocellular carcinomas (HCCs). Here we devised a massive anchored parallel sequencing (MAPS) method using next-generation sequencing to isolate and sequence HBV integrants. Applying MAPS to 40 pairs of HBV–related HCC tissues (cancer and adjacent tissues), we identified 296 HBV integration events corresponding to 286 unique integration sites (UISs) with precise HBV–Human DNA junctions. HBV integration favored chromosome 17 and preferentially integrated into human transcript units. HBV targeted genes were enriched in GO terms: cAMP metabolic processes, T cell differentiation and activation, TGF beta receptor pathway, ncRNA catabolic process, and dsRNA fragmentation and cellular response to dsRNA. The HBV targeted genes include 7 genes (*PTPRJ, CNTN6, IL12B, MYOM1, FNDC3B, LRFN2, FN1*) containing IPR003961 (Fibronectin, type III domain), 7 genes (*NRG3, MASP2, NELL1, LRP1B, ADAM21, NRXN1, FN1*) containing IPR013032 (EGF-like region, conserved site), and three genes (*PDE7A*, *PDE4B*, *PDE11A*) containing IPR002073 (3′, 5′-cyclic-nucleotide phosphodiesterase). Enriched pathways include hsa04512 (ECM-receptor interaction), hsa04510 (Focal adhesion), and hsa04012 (ErbB signaling pathway). Fewer integration events were found in cancers compared to cancer-adjacent tissues, suggesting a clonal expansion model in HCC development. Finally, we identified 8 genes that were recurrent target genes by HBV integration including fibronectin 1 (*FN1*) and telomerase reverse transcriptase (*TERT1*), two known recurrent target genes, and additional novel target genes such as SMAD family member 5 (*SMAD5*), phosphatase and actin regulator 4 (*PHACTR4*), and RNA binding protein fox-1 homolog (*C. elegans*) 1 (*RBFOX1*). Integrating analysis with recently published whole-genome sequencing analysis, we identified 14 additional recurrent HBV target genes, greatly expanding the HBV recurrent target list. This global survey of HBV integration events, together with recently published whole-genome sequencing analyses, furthered our understanding of the HBV–related HCC.

## Author Summary

Integration of the hepatitis B virus (HBV) into the human liver cells was found in most of the related hepatocellular carcinomas (HCCs). Here, taking the recent advances in high-throughput sequencing, we devised an efficient and cost-effective method that we named massive anchored parallel sequencing (MAPS) method, to conduct a global survey of HBV integration events in 40 pairs of HBV–related HCC tissues (cancer and adjacent tissues). We identified 286 unique integration sites (UISs) with precise HBV–Human DNA junctions. We identified a higher number of HBV integration events in cancer adjacent tissues than in HCC tissues, suggesting a clonal expansion process during HCC development. We also found that fibronectin and its related genes (fibronectin type III-like fold domain containing genes) were frequently targeted by HBV. Fibronectin is a protein produced abundantly by the liver cells and also serves as a linker in the extracellular matrix. Our findings might suggest a role for the disruption of fibronectin and associated cellular matrix in HBV related liver cancers. We also identified 14 additional recurrent HBV target genes, greatly expanding the HBV recurrent target list. This study would add significantly to our understanding of HCC development.

## Introduction

Chronic human hepatitis B virus (HBV) infection leads to a range of liver diseases, from chronic hepatitis and liver cirrhosis to hepatocellular carcinoma (HCC). HBV infects approximately 2 billion people worldwide with 350 million suffering from chronic infection. HBV infection causes 500,000 to 1.2 million deaths annually, 320,000 of which are attributable to HCC [1]. Chronic HBV infection has been found to play a causal role in the development HCC from many epidemiological studies [2]. In addition, integrated HBV DNA sequences and episomal HBV genomes have been found in 85–90% of HBV-related HCCs [3].

The role of HBV integration in the development of HCC is rather complicated and yet not fully elucidated. HBV DNA integration was found to be distributed throughout different chromosomal sites in the host genome [4]. These integrations could induce chromosome changes, genome instability, or changes in the expression of human genes. HBV integration events were reported to be associated with chromosome fragile sites or repetitive sequences, and usually followed by local rearrangement, all of which relate to a higher genomic instability [5]. Furthermore, recent studies using genetic approaches and microarray technologies showed that HBV-related HCCs displayed higher rates of chromosomal alterations than HCCs of other risk factors [6]. HBV insertion was found to target the retinoic acid receptor-beta (*RARB*) gene [7] or the human cyclin A2 (*CCNA2*) [8], and generate chimeric oncogenic proteins.

A comprehensive analysis of HBV insertion sites targeting new genes would be desirable and would allow us to understand the mechanism of HCC development and to identify novel therapeutic targets. Towards this comprehensive goal, a moderate high-throughput approach using Alu-PCR approach has identified 68 cellular flanking sequences (seven repetitive or unidentified sequences, 42 cellular genes, and 19 sequences potentially coding for unknown proteins) for HBV integration in the HCC genomes [3]. The targeted genes belong to distinct pathways: calcium signaling related genes, 60s ribosomal protein encoding genes, and platelet derived growth factor and mixed lineage leukemia encoding genes. Recurrent HBV insertions nearby the telomerase reverse transcriptase (*TERT*) have also been reported [3], [9], [10].

Recently, many novel approaches have been developed to identify unique integration sites (UISs) for retrovirus in high-throughput manner using the next-generation sequencing (NGS) technologies including novel nonrestrictive approaches [11]–[13], which avoided preferential amplification and biased identification of UISs for the previous methods that use specific restriction enzymes.

More recently, complete genomic sequencing has been used to identify HBV integration genome-wide, taking advantage of the advance in next-generation sequencing technologies. Sung *et al.* conducted WGS at more than 30 fold coverage for 81 HBV positive HCCs and identified 399 HBV integration events using a criteria of more than 2 read pairs with close mapping positions linking an end of hg19 to an end of HBV, which resulted in the identification of an average of 4.9 HBV integration events per individual patient [14]. In another recently publication, Jiang *et al.* sequenced 4 HCC patients at 80X coverage and identified 255 HBV integration sites, which would generate at about 64 HBV integrations per individual patient [15]. Considering that WGS is still expensive and cost prohibitive for sequencing large number of samples, we have developed, in this study, an alternative method that integrates ligation-mediated PCR with Illumina's paired-end (PE) adapters for amplification and deep sequencing. Here we reported the analysis of 286 unique integration sites (UISs) from 40 pairs of HBV-related HCC and tumor-adjacent tissues, which resulted in the identification of about 7 HBV integrations per individual. We were able to further estimate the abundance of the insertion sites because our method used random fragmentation. Our analysis provided a global profile of HBV DNA integration for HCC. An integrated analysis with recently published whole genome sequencing analyses of HBV-related HCCs [14]–[16] provided us with 14 additional recurrent HBV target genes, thus greatly expanding the HBV recurrent target list.

## Results

### Development of an NGS–based approach for genome-wide analysis of HBV integration

**Figure 1:**
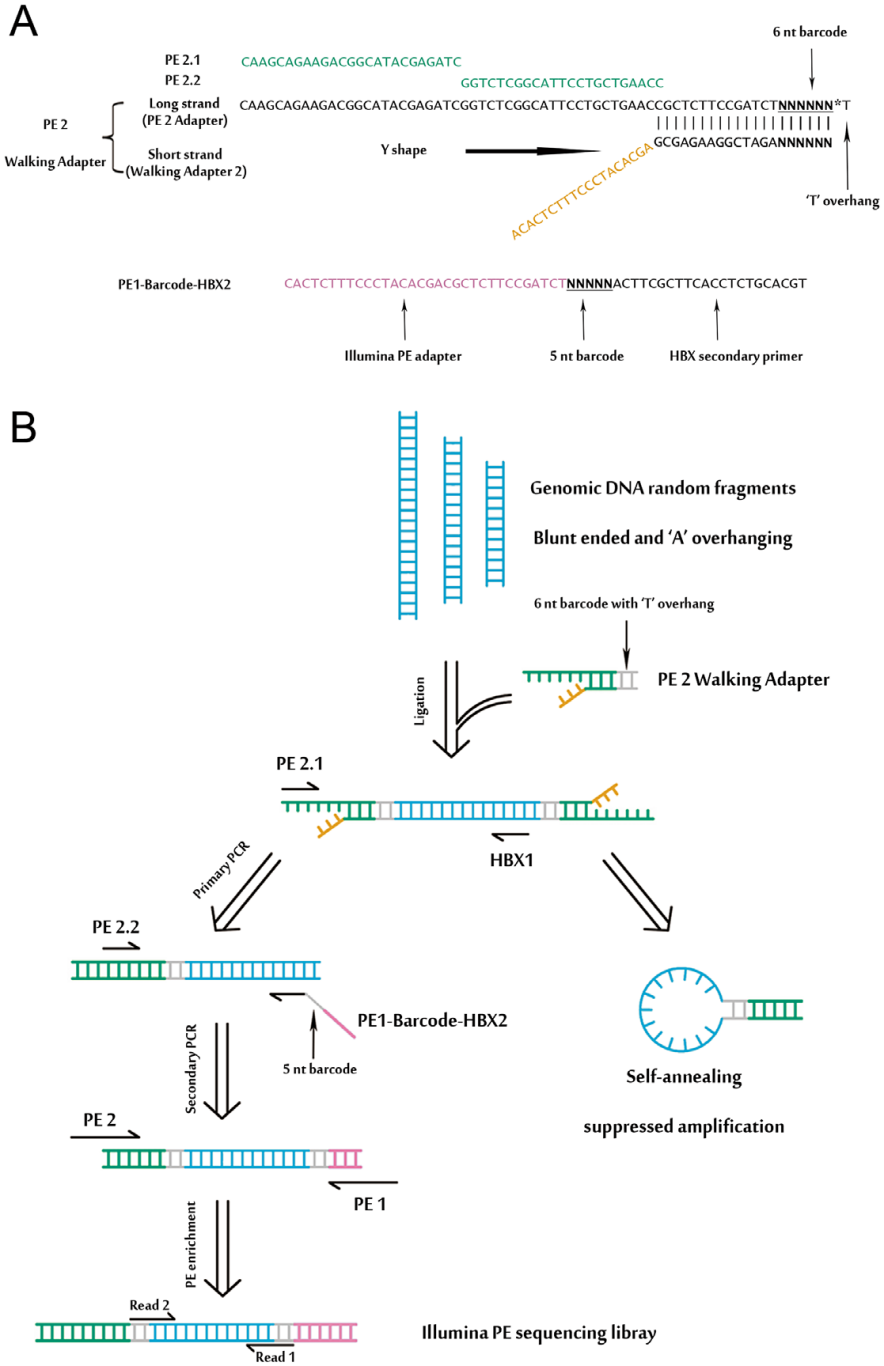
An overview of MAPS. (A) Detailed illustration of the ‘Y’ shaped PE 2 Walking Adapter and the barcoded HBX nested primer. (B) Schematic of the MAPS approach. Adapters and primers are colored in accordance with (A). Detailed primer sequence information is provided in the Table S1, S2 and S3.

We developed a massive anchored parallel sequencing (MAPS) approach to isolate and sequence integrants. The method combines thoughtful designs integrating ligation-mediated PCR with Illumina's PE adapters for amplification and deep sequencing. The MAPS approach stems from the intelligent integration of adapters used in Illumina library preparation with adapters used in LM-PCR, developed for HIV integration site analysis [17]. Illumina uses adapters for flow cell surface annealing, amplification and sequencing while LM-PCR uses adapters (or linkers) as primer matching sites for amplification of unknown nearby sequences. Adopting designing principles for linkers in established genome walking studies [18], we designed a functional adapter building upon Illumina's PE adapter sequence. The PE 2 Walking Adapter was partially complementary and ‘Y’ shaped, with a ‘T’ overhanging on the blunt end (Figure 1A).

A schematic outline of the MAPS is illustrated in Figure 1B, and all MAPS primers are listed in Table S1. Genomic DNA was sheared by sonication, resulting in fragment sizes ranging from 1 kb to 100 bp. The ligation of PE 2 Walking Adapter to the fragments generates an intermediate library with two important characteristics: first, one adapter of the single strand template is identical to Illumina's PE PCR Primer 2 (PE 2); second, the adapter sequence is long enough to suppress unspecific amplification due to single-primer PCR effect [19] during the nested PCR. Targeted sequences were further enriched and full length Illumina PE sequencing adapters were introduced by PE PCR Primer 1 and 2 (PE 1 and PE 2). The library fragments between sizes of 500–600 bp were selected for sequencing.

### DNA bar coding and Illumina PE sequencing of HBV integrations

To make the MAPS approach more efficient, we introduced barcodes to primers for multiplex analysis (Figure 1, Table S2 and S3). In particular, the 6 nt barcode (with a common ‘T’ overhang) was introduced through the barcoded PE 2 Walking Adapters. The 5 nt barcode on Read 1 was introduced by barcoded primers through the secondary PCR.

We applied our MAPS approach in identifying HBV integrations in 40 pairs (cancer and adjacent tissues) of HBV-positive HCCs. According to the previous screen study [3], viral half of chimeric HBV-human DNA was preferentially truncated at a region between 1500–2000 base pairs on the HBV (numbering from the hypothetical EcoRI site of the HBV subtype adw). Therefore, a nested pair of primers in the gene hepatitis B virus x (*HBX*) of HBV was designed based on 4 HBV complete genomes (AY800389.1, AY800390.1, AY800391.1, and AY800392.1) isolated from the same local hospital where we collected the HCC tissues. The nested *HBX* primer (1583–1602), given the preference of viral truncation during integration, was able to generate chimeric HBV-human amplicons suited to the MAPS approach described above. Two negative controls were included, one with the human genomic DNA from an HBV-negative individual, and the other with the above plus purified HBV genomic DNA (i.e. a mixture of HBV DNA with the human genomic DNA, but no integration).

**Figure 2:**
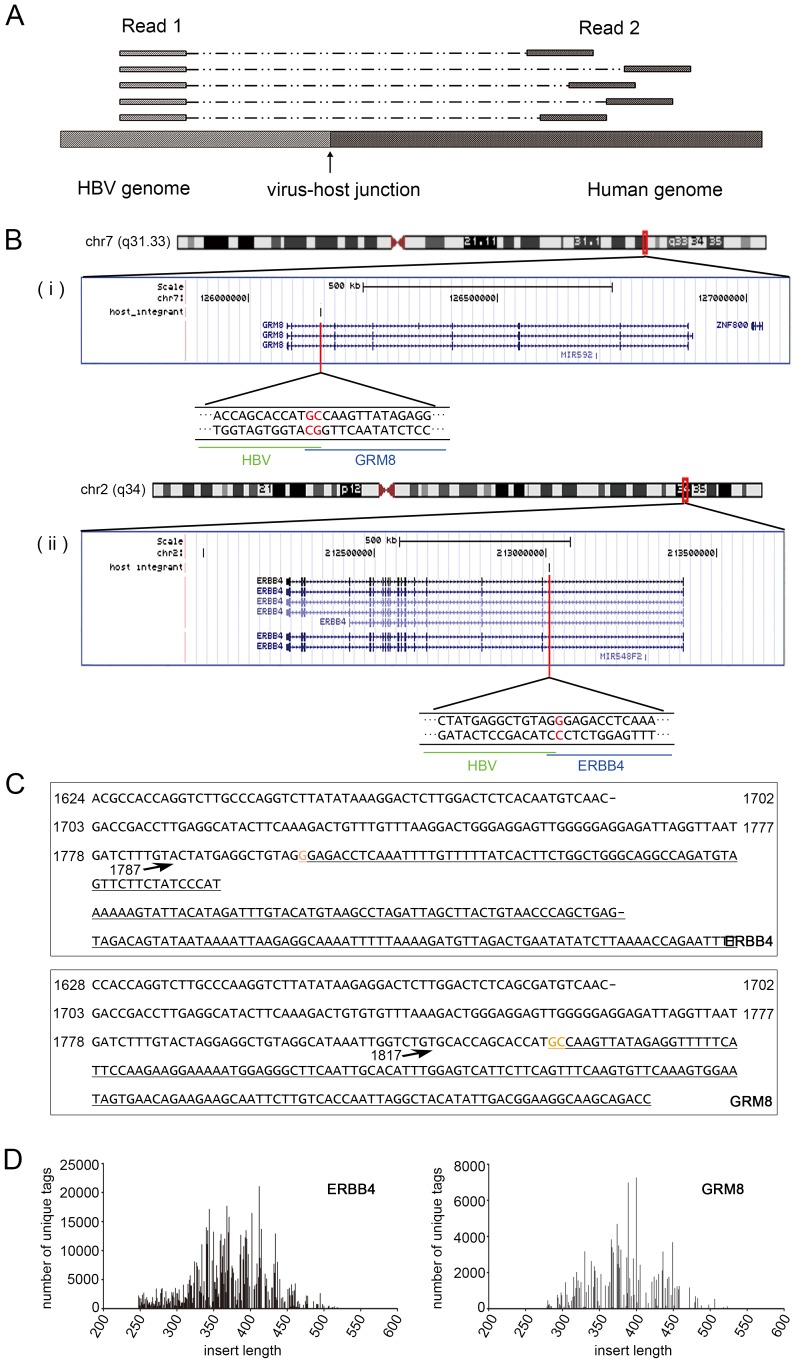
Interpretation of the results of MAPS. (A). A representative mapping of the paired reads from MAPS at integration sites. Read 1 sequences are anchored, since they extend from the fixed HBX nested primer; while the positions of Read 2 sequences vary due to the random fragmentation. (B). Insertion sites illustration in UCSC genome browser. (i) Insertion in ERBB4. (ii) Insertion in GRM8. (C). Integrant sequences of ERBB4 and GRM8. Breakpoint is indicated by arrow. The numbers are HBV genome coordinates, based on the reference of GQ205441. The human gene sequences are underlined. Orange bases indicate the common base between HBV and Human at the junction. (D). Distribution of integrant lengths in the sequenced library of ERBB4 and GRM8 integrations.

After NGS analysis, sequences from both ends were obtained. The Read 1 sequence starts from the same position after the anchored nested *HBX* primer; and the Read 2 sequence, which was obtained from the opposite end, differs in the alignment to the human genome due to the DNA random fragmentation from sonication (Figure 2A). We relied on the DNA shear point deduced in Read 2 to identify putative integrations. To be conservative, integration was determined as authentic if there is a Read 2 sequence present at the corresponding viral-cellular junction.

### Identification of global HBV integrations in the human genome and their distribution features

In the end, we identified 296 HBV DNA integrations (Table S4 and Table 1). 10 of which are paired, identical integrations found in both cancer and their adjacent tissues of the same patient, therefore there were 286 UISs. Among them, 42 sites were found in cancers, while 254 sites were found in adjacent tissues, which is about 6 times more than that found in cancers.

**Table 1:** Summary of HBV targeted genes identified.

Tissue disease state	chr	Insertion orientation	Nubmer of unique end fragments	Number of total reads	Description	Gene symbol (10 kb annotation)*
Adjacent tissue	chrX	+	25	15550	Septin 6	SEPT6
Adjacent tissue	chr16	−	6	4565	RNA binding protein, fox-1 homolog (C. elegans) 1	A2BP1 (RBFOX1)
Adjacent tissue	chr16	+	3	358	RNA binding protein, fox-1 homolog (C. elegans) 1	A2BP1 (RBFOX1)
Adjacent tissue	chr2	−	3	122	Abl interactor 2	ABI2
Adjacent tissue	chr16	+	4	1449	acyl-CoA synthetase medium-chain family member 2A	ACSM2A
Adjacent tissue	chr12	−	23	2062	Acyl-CoA synthetase short-chain family member 3	ACSS3
Adjacent tissue	chr14	−	41	4606	chromosome 14 open reading frame 55	ADAM21
Adjacent tissue	chr4	−	4	811	Alcohol dehydrogenase 1B (class I), beta polypeptide	ADH1B
Adjacent tissue	chr10	−	5	1453	Aldo-keto reductase family 1, member C4 (chlordecone reductase; 3-alpha hydroxysteroid dehydrogenase, type I; dihydrodiol dehydrogenase 4)	AKR1C4
Adjacent tissue	chr4	−	125	61810	Albumin	ALB
Cancer tissue	chr12	−	9	30554	Asparagine-linked glycosylation 10 homolog B (yeast, alpha-1,2-glucosyltransferase)	ALG10B
Adjacent tissue	chrX	−	9	131	Asparagine-linked glycosylation 13 homolog (S. cerevisiae)	ALG13
Adjacent tissue	chr2	−	31	15568	Anaplastic lymphoma kinase (Ki-1)	ALK
Adjacent tissue	chr2	−	18	11626	Alkaline phosphatase, intestinal	ALPI
Adjacent tissue	chr7	−	52	44022	Amphiphysin	AMPH
Adjacent tissue	chr22	+	4	536	Apolipoprotein L, 1	APOL1
Adjacent tissue	chr11	−	16	862	Rho guanine nucleotide exchange factor (GEF) 12	ARHGEF12
Adjacent tissue	chr11	−	6	3607	Rho guanine nucleotide exchange factor (GEF) 12	ARHGEF12
Adjacent tissue	chr8	−	7	6932	ATPase, H+ transporting, lysosomal 38 kDa, V0 subunit d2	ATP6V0D2
Adjacent tissue	chr22	+	18	9882	BCL2-interacting killer (apoptosis-inducing)	BIK
Adjacent tissue	chr7	+	6	658	V-raf murine sarcoma viral oncogene homolog B1	BRAF
Adjacent tissue	chr12	−	20	615	Chromosome 12 open reading frame 40	C12orf40
Adjacent tissue	chr15	+	6	326	DNM1 pseudogene 46	C15orf51
Adjacent tissue	chr4	+	37	2363	C1q and tumor necrosis factor related protein 7	C1QTNF7
Adjacent tissue	chr9	−	9	250	Chromosome 9 open reading frame 93	C9orf93
Adjacent tissue	chr4	+	9	835	coiled-coil domain containing 158	CCDC158
Cancer tissue	chr4	−	130	73841	Cyclin A2	CCNA2
Adjacent tissue	chr4	−	14	9198	Cyclin A2	CCNA2
Adjacent tissue	chr6	−	6	878	Cyclin C	CCNC
Adjacent tissue	chr17	−	11	2684	Cell division cycle 27 homolog (S. cerevisiae)	CDC27
Adjacent tissue	chr7	−	4	2255	cyclin-dependent kinase 13	CDC2L5
Adjacent tissue	chr5	+	18	470	Cadherin 10, type 2 (T2-cadherin)	CDH10
Adjacent tissue	chr16	+	14	6882	Cadherin 8, type 2	CDH8
Adjacent tissue	chr3	+	5	187	Centrosomal protein 63 kDa	CEP63
Adjacent tissue	chr1	+	3	174	Chitinase 3-like 2	CHI3L2
Adjacent tissue	chr12	+	38	693	Citron (rho-interacting, serine/threonine kinase 21)	CIT
Cancer	chr3	−	236	66086	Contactin 6	CNTN6
Adjacent tissue	chr21	−	42	2632	Collagen, type XVIII, alpha 1	COL18A1
Adjacent tissue	chrX	−	45	1849	Collagen, type IV, alpha 6	COL4A6
Adjacent tissue	chr21	−	4	322	Collagen, type VI, alpha 2	COL6A2
Adjacent tissue	chr2	+	95	91537	Carbamoyl-phosphate synthetase 1, mitochondrial	CPS1
Adjacent tissue	chr15	−	28	2082	CTD (carboxy-terminal domain, RNA polymerase II, polypeptide A) small phosphatase like 2	CTDSPL2
Adjacent tissue	chr11	+	9	7279	CUGBP, Elav-like family member 1	CUGBP1
Adjacent tissue	chr10	−	5	92	CUGBP, Elav-like family member 2	CUGBP2
Cancer tissue	chr10	−	298	261343	Cytochrome P450, family 2, subfamily C, polypeptide 8	CYP2C8
Adjacent tissue	chr10	+	29	6742	Cytochrome P450, family 2, subfamily C, polypeptide 8	CYP2C8
Adjacent tissue	chr11	+	5	168	Discs, large homolog 2, chapsyn-110 (Drosophila)	DLG2
Adjacent tissue	chr8	−	3	46	Dihydropyrimidinase	DPYS
Adjacent tissue	chr6	−	61	5013	Ectonucleotide pyrophosphatase/phosphodiesterase 1	ENPP1
Adjacent tissue	chr1	+	4	60	Epidermal growth factor receptor pathway substrate 15	EPS15
Adjacent tissue	chr2	+	301	792607	V-erb-a erythroblastic leukemia viral oncogene homolog 4 (avian)	ERBB4
Cancer	chr16	+	16	6616	Endoplasmic reticulum to nucleus signaling 2	ERN2
Adjacent tissue	chr1	+	58	7369	Estrogen-related receptor gamma	ESRRG
Adjacent tissue	chr4	−	15	8951	Ellis van Creveld syndrome	EVC
Adjacent tissue	chr17	+	14	10665	Family with sequence similarity 117, member A	FAM117A
Adjacent tissue	chr3	−	25	1844	family with sequence similarity 162, member A	FAM162A
Adjacent tissue	chr5	+	4	957	F-box and leucine-rich repeat protein 17	FBXL17
Adjacent tissue	chr5	+	9	196	F-box and leucine-rich repeat protein 7	FBXL7
Adjacent tissue	chr4	−	23	318	KIAA1727 protein	FHDC1
Adjacent tissue	chr18	+	6	5412	Formin homology 2 domain containing 3	FHOD3
Adjacent tissue	chr2	−	169	36268	Fibronectin 1	FN1
Adjacent tissue	chr2	−	9	1054	Fibronectin 1	FN1
Adjacent tissue	chr2	−	47	631	Fibronectin 1	FN1
Adjacent tissue	chr2	−	8	301	Fibronectin 1	FN1
Cancer tissue	chr2	−	58	33209	Fibronectin 1	FN1
Adjacent tissue	chr17	+	9	30405	Fructosamine-3-kinase-related protein	FN3KRP
Adjacent tissue	chr3	+	89	15775	Fibronectin type III domain containing 3B	FNDC3B
Adjacent tissue	chr4	+	47	30826	Fraser syndrome 1	FRAS1
Adjacent tissue	chr4	+	32	1331	Follistatin-like 5	FSTL5
Adjacent tissue	chr14	−	3	35	exonuclease 3′-5′ domain containing 2	GALNTL1
Adjacent tissue	chr7	−	3	2771	UDP-N-acetyl-alpha-D-galactosamine:polypeptide N-acetylgalactosaminyltransferase-like 5	GALNTL5
Cancer tissue	chr5	−	30	2690	Glucosamine-6-phosphate deaminase 1	GNPDA1
Adjacent tissue	chr13	−	4	1057	Glypican 6	GPC6
Adjacent tissue	chr4	−	5	125	Glutamate receptor, ionotropic, delta 2	GRID2
Adjacent tissue	chr7	+	156	127769	Glutamate receptor, metabotropic 8	GRM8
Adjacent tissue	chr12	+	22	453	Glycogen synthase 2 (liver)	GYS2
Adjacent tissue	chr11	+	6	22742	Mps one binder kinase activator-like 2	HCCA2
Adjacent tissue	chr6	−	6	3624		HCG27
Adjacent tissue	chr6	+	6	3221	Histone cluster 1, H2bd	HIST1H2BD
Adjacent tissue	chr19	−	6	256	Histocompatibility (minor) HA-1	HMHA1
Adjacent tissue	chr10	+	25	1214	Heparanase 2	HPSE2
Adjacent tissue	chr3	+	18	504	Histidine-rich glycoprotein	HRG
Adjacent tissue	chr5	+	5	568	Interleukin 12B (natural killer cell stimulatory factor 2, cytotoxic lymphocyte maturation factor 2, p40)	IL12B
Adjacent tissue	chr3	+	5	2776	Interleukin 1 receptor accessory protein	IL1RAP
Adjacent tissue	chr10	−	4	2968	Interleukin 2 receptor, alpha	IL2RA
Adjacent tissue	chr6	+	5	431	Interphotoreceptor matrix proteoglycan 1	IMPG1
Adjacent tissue	chr19	−	10	3440	Inositol 1,4,5-trisphosphate 3-kinase C	ITPKC
Adjacent tissue	chr17	+	124	1293	K(lysine) acetyltransferase 2A	KAT2A
Adjacent tissue	chr14	+	8	291	KIAA0317	KIAA0317
Adjacent tissue	chr3	+	8	636	Kelch-like 6 (Drosophila)	KLHL6
Adjacent tissue	chr6	−	9	304	Lin-28 homolog B (C. elegans)	LIN28B
Cancer tissue	chr9	−	53	4292	Leucine rich repeat and Ig domain containing 2	LINGO2
Adjacent tissue	chr7	+	45	1917	Limb region 1 homolog (mouse)	LMBR1
Adjacent tissue	chr12	−	4	119	WAS protein family homolog 1 pseudogene	LOC100288778
Adjacent tissue	chr5	+	7	10522	Hypothetical protein LOC285627	LOC285627
Adjacent tissue	chr17	−	5	246		LOC644172
Adjacent tissue	chr6	+	62	1101	Leucine rich repeat and fibronectin type III domain containing 2	LRFN2
Adjacent tissue	chr2	+	45	703	Low density lipoprotein-related protein 1B (deleted in tumors)	LRP1B
Adjacent tissue	chr15	−	5	582	Leucine-rich repeat kinase 1	LRRK1
Adjacent tissue	chr4	+	14	14654	Mastermind-like 3 (Drosophila)	MAML3
Adjacent tissue	chr1	+	6	2023	Mannan-binding lectin serine peptidase 2	MASP2
Adjacent tissue	chr3	−	3	48	Muscleblind-like (Drosophila)	MBNL1
Adjacent tissue	chr6	−	43	3090	Mediator complex subunit 23	MED23
Adjacent tissue	chr19	+	12	9041	Meis homeobox 3	MEIS3
Adjacent tissue	chr7	+	5	68	microRNA 182	MIR182
Adjacent tissue	chr6	+	4	90	microRNA 206	MIR206
Adjacent tissue	chr7	+	11	631	Myeloid/lymphoid or mixed-lineage leukemia 3	MLL3
Cancer tissue	chr19	−	80	4468	Myeloid/lymphoid or mixed-lineage leukemia 4	MLL4
Cancer	chr2	−	26	840	Melanoregulin	MREG
Adjacent tissue	chr5	+	16	650	Myosin X	MYO10
Adjacent tissue	chr18	+	31	17222	Myomesin 1, 185 kDa	MYOM1
Cancer tissue	chr7	+	192	76342	long intergenic non-protein coding RNA 174	NCRNA00174
Adjacent tissue	chr11	+	3	129	NEL-like 1 (chicken)	NELL1
Adjacent tissue	chr6	−	127	123237	Na+/K+ transporting ATPase interacting 2	NKAIN2
Adjacent tissue	chr10	−	88	73961	Neuregulin 3	NRG3
Adjacent tissue	chr2	−	43	31154	Neurexin 1	NRXN1
Adjacent tissue	chr3	−	6	7428	Poly (ADP-ribose) polymerase family, member 9	PARP9
Adjacent tissue	chr2	+	4	84	Phosphodiesterase 11A	PDE11A
Adjacent tissue	chr1	−	4	278	Phosphodiesterase 4B, cAMP-specific (phosphodiesterase E4 dunce homolog, Drosophila)	PDE4B
Adjacent tissue	chr8	+	8	10926	Phosphodiesterase 7A	PDE7A
Adjacent tissue	chr6	+	14	442	enoyl-CoA delta isomerase 2	PECI
Adjacent tissue	chr14	+	4	1864	Placental growth factor, vascular endothelial growth factor-related protein	PGF
Cancer	chr5	−	289	3293	Protein geranylgeranyltransferase type I, beta subunit	PGGT1B
Adjacent tissue	chr1	−	14	216	Phosphatase and actin regulator 4	PHACTR4
Adjacent tissue	chr1	−	11	491	Phosphatase and actin regulator 4	PHACTR4
Cancer tissue	chr12	−	54	6030	Polyhomeotic homolog 1 (Drosophila)	PHC1
Cancer	chr3	−	107	7765	Phosphoinositide-3-kinase, catalytic, beta polypeptide	PIK3CB
Adjacent tissue	chr20	−	7	1858	Phospholipase C, beta 1 (phosphoinositide-specific)	PLCB1
Adjacent tissue	chr1	−	12	5993	Phospholipase D family, member 5	PLD5
Adjacent tissue	chr7	−	29	10098	Plexin A4	PLXNA4
Adjacent tissue	chr12	+	6	256	Protein phosphatase 1H (PP2C domain containing)	PPM1H
Adjacent tissue	chr8	+	20	700	Protein kinase, DNA-activated, catalytic polypeptide	PRKDC
Adjacent tissue	chr17	+	7	2875	Proteasome (prosome, macropain) 26S subunit, non-ATPase, 12	PSMD12
Adjacent tissue	chr11	+	43	9668	Protein tyrosine phosphatase, receptor type, J	PTPRJ
Adjacent tissue	chr17	+	18	714	RAB5C, member RAS oncogene family	RAB5C
Adjacent tissue	chr17	−	6	320	Retinoic acid induced 1	RAI1
Adjacent tissue	chr17	−	77	9045	Retinoic acid receptor, alpha	RARA
Cancer	chr10	−	306	96735	Hypothetical protein LOC220980	RASSF4
Adjacent tissue	chr10	−	4	127	Retinol binding protein 4, plasma	RBP4
Adjacent tissue	chr6	+	39	2877	RALBP1 associated Eps domain containing 1	REPS1
Adjacent tissue	chrX	+	4	1297	Regucalcin (senescence marker protein-30)	RGN
Adjacent tissue	chr17	+	11	4735	Rho GTPase activating protein 44	RICH2
Adjacent tissue	chr15	+	3	120	S phase cyclin A-associated protein in the ER	SCAPER
Adjacent tissue	chr6	+	138	23599	Sex comb on midleg-like 4 (Drosophila)	SCML4
Adjacent tissue	chr2	+	4	1532	SEC14 and spectrin domains 1	SESTD1
Adjacent tissue	chr8	+	3	554	Secreted frizzled-related protein 1	SFRP1
Adjacent tissue	chr14	+	77	795	surfactant associated 3	SFTA3
Adjacent tissue	chr5	+	6	579	Superkiller viralicidic activity 2-like 2 (S. cerevisiae)	SKIV2L2
Adjacent tissue	chr12	−	27	22758	Solute carrier family 2 (facilitated glucose transporter), member 13	SLC2A13
Adjacent tissue	chr3	+	26	14482	Sarcolemma associated protein	SLMAP
Adjacent tissue	chr4	−	6	8441	SMAD family member 1	SMAD1
Cancer	chr5	+	570	20953	SMAD family member 5	SMAD5
Adjacent tissue	chr5	+	31	310	SMAD family member 5	SMAD5
Cancer	chr5	+	30	1124	SMAD family member 5	SMAD5
Adjacent tissue	chr2	−	14	2397	Son of sevenless homolog 1 (Drosophila)	SOS1
Adjacent tissue	chr12	+	16	7109	SRY (sex determining region Y)-box 5	SOX5
Adjacent tissue	chr11	+	4	56	Spondin 1, extracellular matrix protein	SPON1
Adjacent tissue	chr1	+	7	8119	Single stranded DNA binding protein 3	SSBP3
Adjacent tissue	chr8	+	11	4638	Suppression of tumorigenicity 18 (breast carcinoma) (zinc finger protein)	ST18
Adjacent tissue	chr1	−	17	1196	Syntaxin 6	STX6
Adjacent tissue	chr5	+	7	469	Synaptic vesicle glycoprotein 2C	SV2C
Adjacent tissue	chr8	+	42	8798	Transforming, acidic coiled-coil containing protein 1	TACC1
Adjacent tissue	chr8	+	16	4216	TAF2 RNA polymerase II, TATA box binding protein (TBP)-associated factor, 150 kDa	TAF2
Cancer tissue	chr5	−	382	46659	Telomerase reverse transcriptase	TERT
Cancer	chr5	−	86	2724	Telomerase reverse transcriptase	TERT
Cancer tissue	chr5	−	63	3789	Telomerase reverse transcriptase	TERT
Cancer	chr5	−	230	2853	Telomerase reverse transcriptase	TERT
Cancer tissue	chr5	−	270	7445	Telomerase reverse transcriptase	TERT
Adjacent tissue	chr5	−	5	259	Telomerase reverse transcriptase	TERT
Cancer tissue	chr5	−	392	47491	Telomerase reverse transcriptase	TERT
Adjacent tissue	chr5	−	55	2941	Telomerase reverse transcriptase	TERT
Adjacent tissue	chr12	+	47	3611	Transmembrane protein 117	TMEM117
Adjacent tissue	chr12	+	3	105	Transmembrane protein 132B	TMEM132B
Adjacent tissue	chr15	−	11	6194	Transmembrane protein 87A	TMEM87A
Adjacent tissue	chr1	+	7	412	Ubiquitin specific peptidase 33	USP33
Adjacent tissue	chr2	+	4	225	Xin actin-binding repeat containing 2	XIRP2
Adjacent tissue	chr11	−	3	244	Zinc finger and BTB domain containing 16	ZBTB16
Adjacent tissue	chr16	−	81	13492	Zinc finger protein 1 homolog (mouse)	ZFP1
Adjacent tissue	chr9	+	12	5969	Zinc finger protein 618	ZNF618
Adjacent tissue	chr7	−	9	370	Zinc finger protein 804B	ZNF804B

We randomly picked 28 putative insertions and used a standardized nested-PCR assay, we confirmed 27 out of 28 putative insertions (96%) (Table S5). Our confirmation rate is similar to what Sung *et al.* reported for the HBV insertions from whole genome sequencing, which they randomly selected 32 breakpoints at the 6 affected genes for PCR analysis in 22 samples and were able to successfully validate 82% of these integration sites [14]. To further validate those putative sites, we sequenced two amplified integrant PCR products (HBV integration in ERBB4 and GRM8) by the Sanger method. BLAST analysis revealed that both sequences extend from the HBX gene to the identified sites in the human genome (Figure 2B and 2C). We also examined the length of DNA fragments in the sequencing library. The lengths of the integrants (without the adapter and barcode sequences) clustered around 350–450 bp (Figure 2D).

We annotated insertions the nearest RefSeq genes within 10 kb from the HBV-Human junction points (Table S4) and this annotation was used in the other analyses in this manuscript. However, we also provided the annotations to within 150 kb in the Table S4 as previous studies showed that the HBV genome includes enhancer elements capable of activating promoters even 150 kb away, in a position and orientation independent manner [9], [20].

We compared the genes targeted by HBV from our study to three recently published studies. Fujimoto *et al.* only identified 6 genes (*ADAM5P, FRAS1, THRB, HNF1A, FAM18B2, TTLL9*) in addition to *TERT* (their supplementary table 13) from 11 HBV-related HCCs in 23 integration events [16]. Only the *TERT* gene is in common with our HBV targeted gene list. Sung *et al.* identified 399 HBV integration events from 81 HBV positive HCCs (from their supplementary table S3). As they did not indicate what distance they allowed from the HBV integration to the start or end site of genes, we re-annotated their list with the same criterion we used in our annotation to within 10 kb of genes, and then performed the comparison. Jiang *et al.* reported the identification of 255 HBV integration events in three HBV positive HCCs [15]. We also re-annotated Jiang *et al.*'s list to within 10 kb of the gene for the comparison. Comparing our list (Table 1) to Sung *et al.*'s list, we found that there are 9 genes in common (Table 2). Comparing our list to Jiang *et al.*'s list, we found that they are four genes in common. Two genes, *FN1* (fibronectin 1) and *MLL4* (Myeloid/lymphoid or mixed-lineage leukemia 4) were found in all three studies (Table 2). Except for *FN1* and *MLL4*, there were no additional common genes between the Sung *et al.*'s list and Jiang *et al.*'s list.

**Table 2:** Common HBV targeted genes among the three studies.

Our study	Sung et al.	Jiang et al.	Description
ADH1B	ADH1B		Alcohol dehydrogenase 1B (class I), beta polypeptide
CPS1	CPS1		Carbamoyl-phosphate synthetase 1, mitochondrial
ESRRG	ESRRG		Estrogen-related receptor gamma
FN1	FN1	FN1	Fibronectin 1
LRFN2	LRFN2		Leucine rich repeat and fibronectin type III domain containing 2
MLL4	MLL4	MLL4	Myeloid/lymphoid or mixed-lineage leukemia 4
MYOM1	MYOM1		Myomesin 1, 185 kDa
RAI1	RAI1		Retinoic acid induced 1
TERT	TERT		Telomerase reverse transcriptase
CTDSPL2		CTDSPL2	CTD (carboxy-terminal domain, RNA polymerase II, polypeptide A) small phosphatase like 2
LRP1B		LRP1B	Low density lipoprotein-related protein 1B (deleted in tumors)

We also checked all the HBV integration events against the miRNA clusters using the genomic coordinates of all miRNAs in the miRBase (www.mirbase.org). We found one integration event at ChrX:108297754 that maps 18 nucleotides upstream of miRNA has-mir-6087. Has-mir-6087 was recently identified in human ES cells [21].

**Figure 3:**
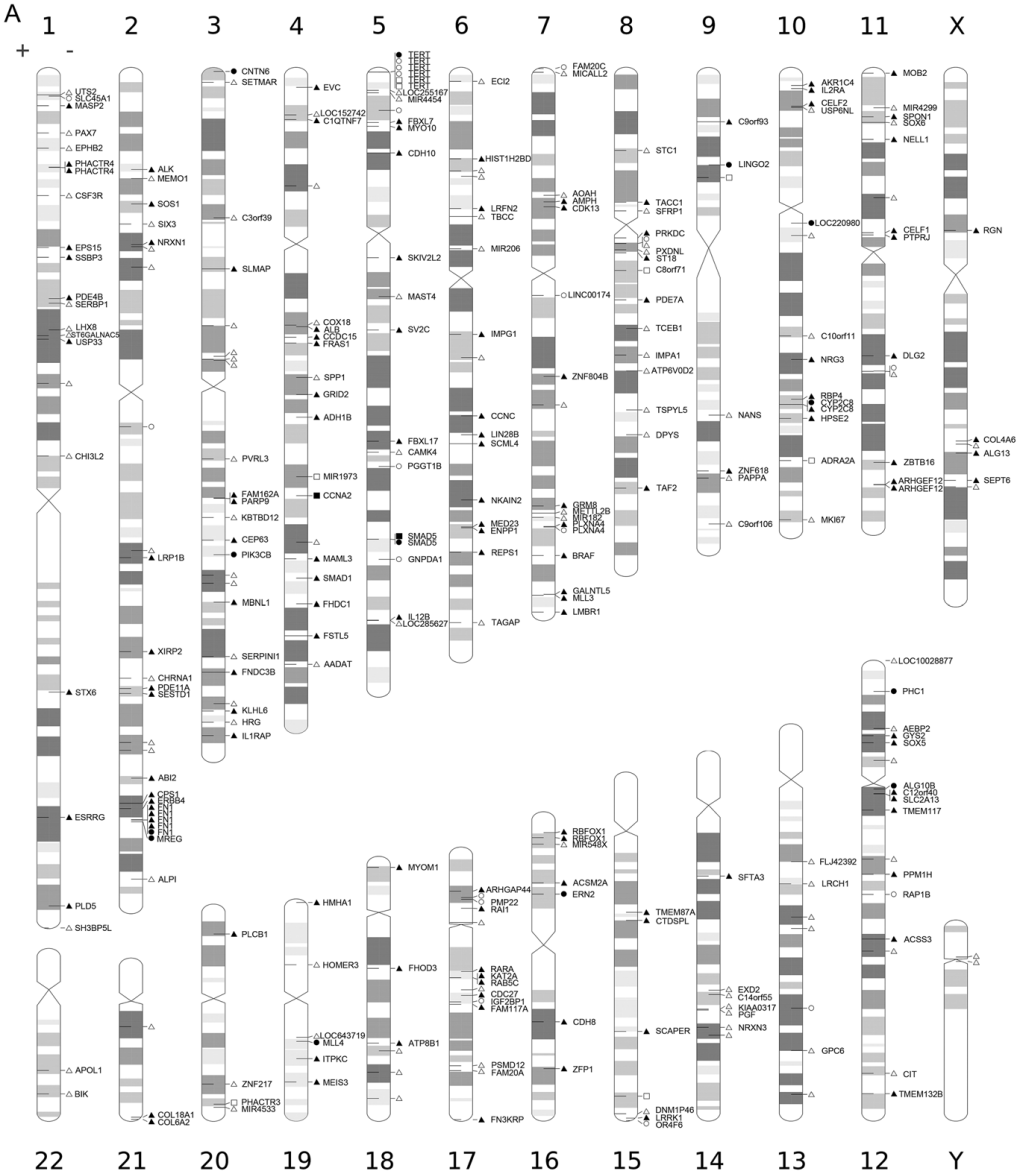
Distribution of the identified UISs. Locations of UISs in the human genome. Circles indicate the UISs found only in cancer tissue, triangles represent UISs only found in the adjacent tissue, and rectangles mean UISs found in both. The nearest gene in the vicinity of UIS was annotated, and the filled shapes marks UISs that integrate in the transcript unit.

**Figure 4:**
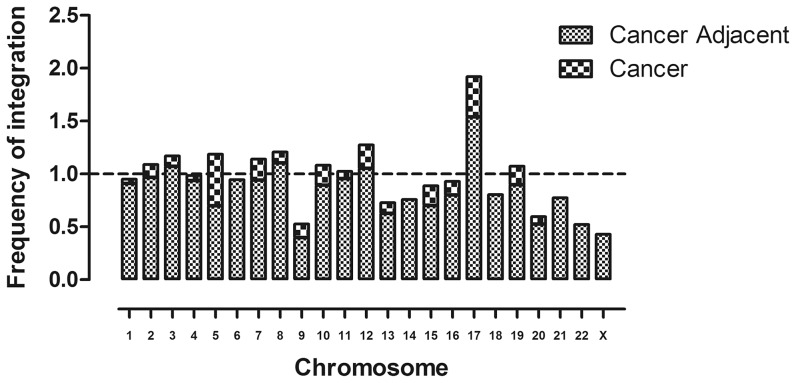
Number of UISs per chromosome. A frequency of 1 (represented by the dotted line) stands for the same level of integration as the random data, i.e., this chromosome is neither favored nor disfavored for HBV integration.

We also mapped the position of each UIS to chromosomes and generated a superimposed diagram illustrating integration of UISs to within cytogenetic bands of the human genome (Figure 3). To analyze whether the HBV integrations had any preferences over transcript units and individual chromosomes, a set of 8015 computer-simulated random integrations was generated as the control. We calculated the frequency of HBV integration within each chromosome, showing that chromosome 17 was favored target for HBV integration (Figure 4). 60.5% of the HBV integrations (178/294) landed in RefSeq genes (Table S4). This is significantly different from the result of the random dataset, of which only 42.68% landed in genes reflecting the feature of the human genomes (Pearson's chi-squared test, *P*<0.0001).

### Functional annotations of the HBV targeted genes

To understand the function of the genes targeted by HBV, we performed Gene Ontology (GO) analysis using the GO miner program. We found that they are enriched in 3 GO terms related to cAMP metabolic processes and several GO terms related to T-cell differentiation and activation involved in immune response (Table S6). Interestingly, GO terms related to GO:0034661 ncRNA catabolic process, GO:0031050 dsRNA fragmentation, GO:0070918 production of small RNA involved in gene silencing by RNA, and GO:0071359 cellular response to dsRNA were also enriched (Table S6). These suggest that the HBV targeted host genes related to responding, or to defending or eliminating the virus such as the immune response and degradation of RNAs. We also found that GO terms related to TGF beta receptor pathway were enriched (Table S6).

To understand whether HBV targeted genes are enriched with particular protein domains, we used the DAVID (Database for Annotation, Visualization and Integrated Discovery) Functional Annotation tool to analyze the combined list of HBV targeted genes (Table 1). The enriched INTERPRO terms with EASE score, which is a modified Fisher Exact P-Value, <0.05, were shown is Table S7. We found that 7 genes (*PTPRJ, CNTN6, IL12B, MYOM1, FNDC3B, LRFN2, FN1*) contain the IPR003961 (Fibronectin, type III domain), 7 genes (*NRG3, MASP2, NELL1, LRP1B, ADAM21, NRXN1, FN1*) with the IPR013032 (EGF-like region), conserved site, and three genes (*PDE7A*, *PDE4B*, *PDE11A*) containing the IPR002073 (3′, 5′-cyclic-nucleotide phosphodiesterase) (Table S7). Mapping the HBV targeted genes to the KEGG pathways, we found that they were enriched in several KEGG pathways including hsa04512 (ECM-receptor interaction), hsa04510 (Focal adhesion), and hsa04012 (ErbB signaling pathway) (Table S8).

### Semi-quantification of HBV insertion sites based on tags covering the integration sites

**Figure 5:**
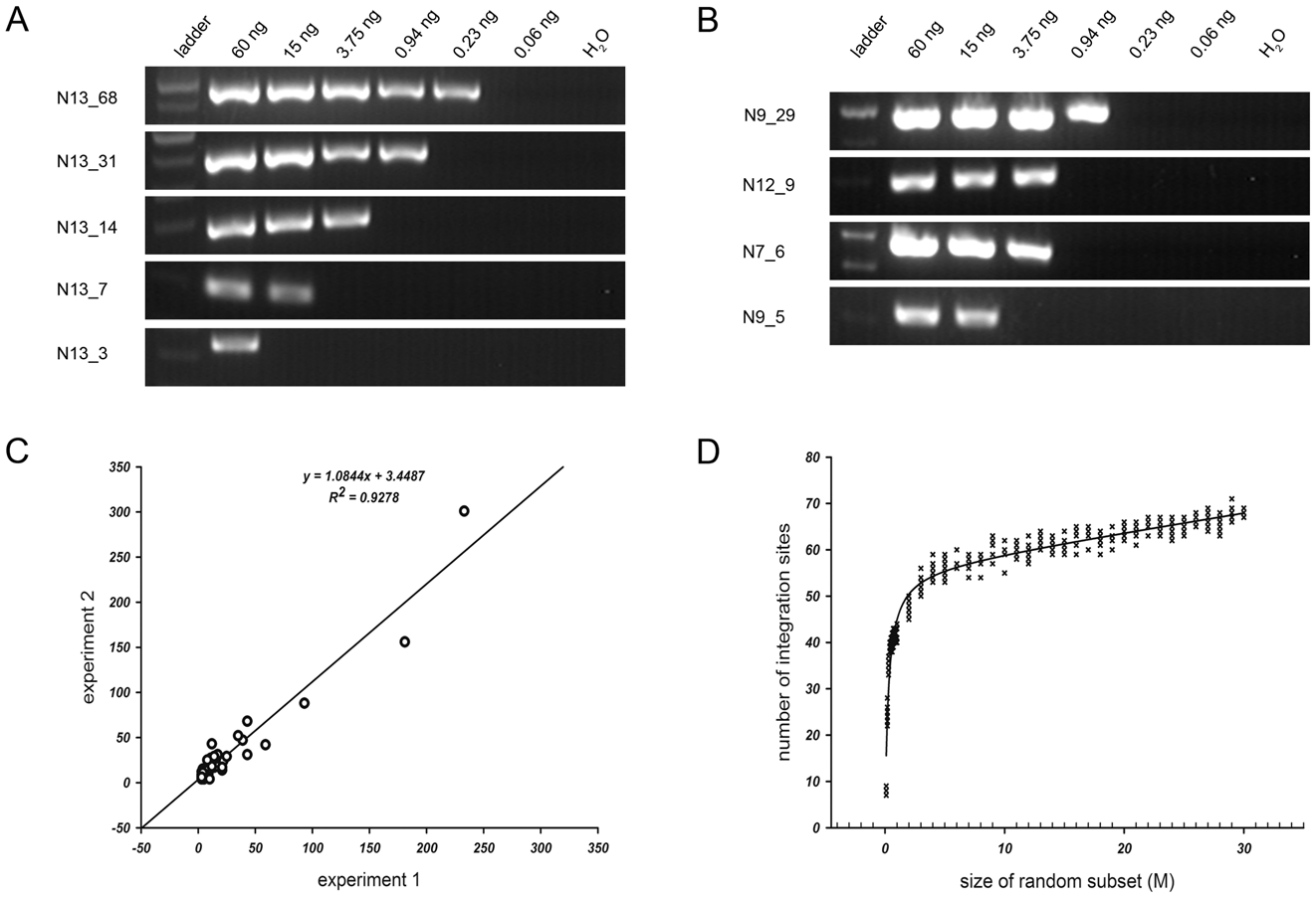
Quantitative feature of MAPS. (A) Semi-quantitative PCR results in HCC DNA sample N13. Each panel represents different integration frequencies, judged by the number of unique tags identified by MAPS, as indicated by the number followed by the sample name on the left column labels. The amounts of genomic DNA used for PCR were indicated at the top of the lanes. (B) Semi-quantitative PCR results in multiplexed DNA samples. The left column indicates the HCC DNA sample identifications and the number of unique tags identified. (C) Correlation co-efficiency (R^2^ = 0.9278) of the numbers of unique tags covering the common insertions retrieved between two technical replicate runs. (D) Rarefaction analysis of integration sites recovered in MAPS.

The abundance of the UISs was quantified by the count of different shear points near the junction, a method that was also utilized by Gillet *et al.* [11]. In addition, we tested the quantitative potential of the UISs abundance by a serial dilution PCR, and found that the number of unique tags covering an integration site correlated with the abundance of the integration determined by serial dilution PCR, both for an individual sample (Figure 5A) and across different samples (Figure 5B).

To examine technical reproducibility of the quantification, we compared duplicate results using same DNA samples but sheared separately. The correlation co-efficiency of the number of unique tags covering the common integration sites, even between two technical replicate experiments with slight modifications of primers used (one with the single 5 nt barcode and the other with the paired barcodes), was very high (R^2^ = 0.9278; Figure 5C). We also randomized the total reads into 39 subsets and plotted a rarefaction curve showing the number of putative integrations identified with accumulating subsets. The curve plateaus in Figure 5D suggested that further sequencing would not yield many additional integration sites. The reliability of quantification was also reflected by the consistency in the number of unique tags covering the opposite integration junctions from the same integrations (data not shown).

### Fewer UISs were identified in HCC comparing to adjacent tissues supporting the theory of clonal expansion in HCC development

**Figure 6:**
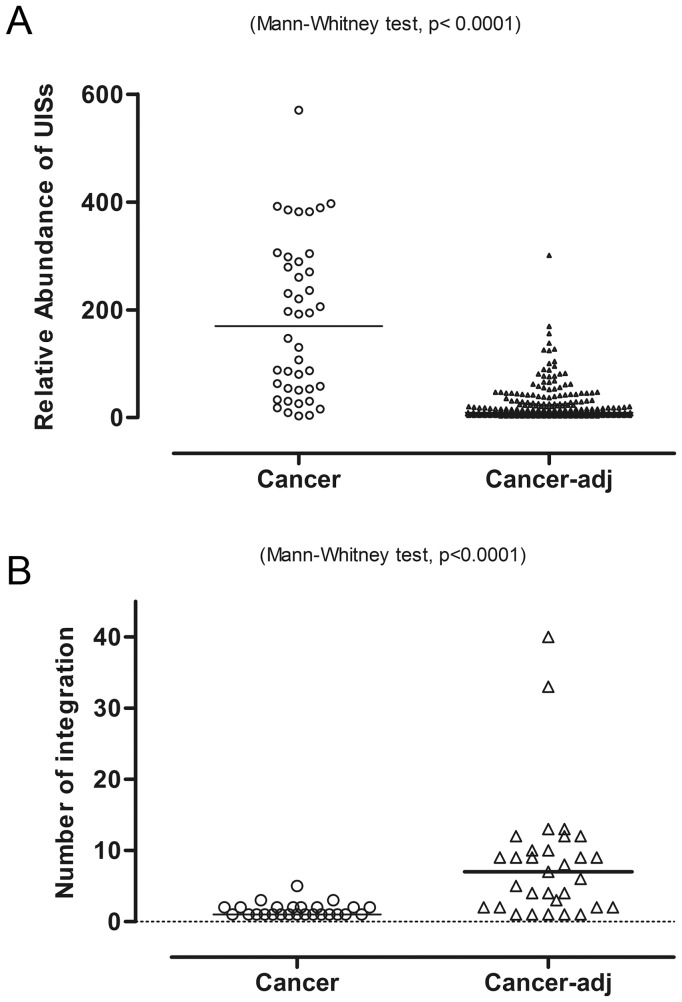
More clonal expansion but fewer insertions were found in cancer than in the adjacent tissue. (A) We counted the number of unique tags to quantify the relative abundance of each UIS. The bar is the median of the number of unique tags. (B) Number of HBV integrations identified in cancer and adjacent tissue. The bar represents the median of the number of integrations.

As each integration event provides a unique genetic marker for a cell, we can assess the clonal expansion of HBV integrated hepatocytes using UISs. We found that the cancer tissues showed clonal expansion compared to tumor-adjacent tissues, as judged by the number of shear sites near individual integration (Figure 6, Mann–Whitney test, *p*<0.0001). Furthermore, the number of UISs (median = 1) found in cancer was less than that of the tumor-adjacent tissue (median = 7, Figure 6B, Mann–Whitney test, *p*<0.0001), consistent with the results from previous studies [3], [22]. Interestingly, there was no difference in the UIS abundance between clones inserted in the vicinity of genes and clones that were not (Mann–Whitney test, *p* = 0.8576) (Data not shown).

### Recurrent HBV integration into 8 human host genes including TERT and FN1 genes

We annotated the 286 UISs with RefSeq genes and found that 8 genes were targeted by HBV integration for more than once (Table 3), including *ARHGEF12* (Rho guanine nucleotide exchange factor (GEF) 12), *CYP2C8* (cytochrome P450, family 2, subfamily C, polypeptide 8), *FN1* (fibronectin 1), *PHACTR4* (Phosphatase and actin regulator 4), *PLXNA4* (Plexin A4), *RBFOX1* [RNA binding protein, fox-1 homolog (C. elegans) 1], *SMAD5* (SMAD family member 5), and *TERT* (telomerase reverse transcriptase).

**Table 3:** Recurrent HBV targeted genes identified by the MAPS approach.

Sample ID	Tissue disease state	chr	insertion orientation	number of unique end fragments	number of total reads	Gene symbol	Description	transcript direction	junction nucleotide position	read covering the junction point (Underlined for HBV)
N23	Adjacent tissue	chr11	−	16	862	ARHGEF12	Rho guanine nucleotide exchange factor (GEF) 12	+	120259620	TTTATTTTGCGACAGAGTTTTGCTCTTGTTTCCCAGGCTGCCTACAGCCTCCTAGTACAAAGACCTTTAACCTCATCTCCTCCCCCAA
N22	Adjacent tissue	chr11	−	6	3607	ARHGEF12	Rho guanine nucleotide exchange factor (GEF) 12	+	120345441	CTATGACAAAGGGAAGAGGAGAGAACCATTCTCACTCATGCACTGATTAGTTGCATGGTGCTGGTGAACAGACCAATTTATGCCTACA
C23	Cancer tissue	chr10	−	298	261343	CYP2C8	Cytochrome P450, family 2, subfamily C, polypeptide 8	−	96804432	TCTCACAGTTTCCTGGCCCTGTAAAGTGTCGCTCATAGTACAAAGACCTTTAACCTCATCTCCTACCCCAACTCCTCCCACTCATTAA
N102	Adjacent tissue	chr10	+	29	6742	CYP2C8	Cytochrome P450, family 2, subfamily C, polypeptide 8	−	96817072	CCATATATCACCCAATTTAAAGTGTACACTTTAGTACATAAATCTTTAACCTAATCTCCTCCCCCAACTCCTCCCAGTCCTTAAACCA
N113	Adjacent tissue	chr2	−	169	36268	FN1	Fibronectin 1	−	216249015	TTTATAGCAGCATGTAAATCACATCTTCTTGGTGAACAGACCAATTTATGCCTACAGCCTCCTAGTACAAA
N19	Adjacent tissue	chr2	−	9	1054	FN1	Fibronectin 1	−	216252251	AAGACATTATCCCAGACAAATTACAATGCTGATAATTCCTCGTGAATTGACAAGAAAAAGTTGCATGGTGCTGGTGAACAGACCAATT
N118	Adjacent tissue	chr2	−	47	631	FN1	Fibronectin 1	−	216254297	GGGCTTGTAATCCCAGCACTTTGGGAGGCCGAGGAGGGTGGAGCACTTGAGTACTGGTGAACAGACCAATT
N106	Adjacent tissue	chr2	−	8	301	FN1	Fibronectin 1	−	216274425	GGCTGTGATTTCGGTCACAGATTCAGAAGTGGCCACAAGAAGTACAAAGATCATTAACCTAATCTCCTCCCCCAACTCCTACCAGTCT
C25	Cancer tissue	chr2	−	58	33209	FN1	Fibronectin 1	−	216293206	TTCATATTCAGCTCTGGTGCTGCAAGGTATTACAGCCTCCTAGTACAAAGACCTTTAACCTAATCTCCTCCCCCAACTCCTCCCAGTC
N125	Adjacent tissue	chr1	−	14	216	PHACTR4	Phosphatase and actin regulator 4	+	28785619	TTTTCATGTAACAGAGGAAGCAGACCAGCCCACTACAGTGTGGCATGGTGCTGGTGAACAGACCAATTTAT
N23	Adjacent tissue	chr1	−	11	491	PHACTR4	Phosphatase and actin regulator 4	+	28819591	AAAAGCTCCGAGATGGAGGTTCATGAAGAGAGCAATCGAACACACCAATTTATGCCTACAGCCTCCTAGTACAAAGACCTTTAACCTC
N9	Adjacent tissue	chr7	−	29	10098	PLXNA4	Plexin A4	−	132158848	AAACAAAACAAAACAAAAAAAAACAGGGCAATAAACGGTGCACAGACCAATTTATGCCTACAGCCTCCTAGTACAAAGATCATTAACC
C116	Cancer tissue	chr7	−	4	521	PLXNA4 upstream	within 150 kb of Plexin A4	−	132358062	AAGACTTCTCCTTTGGGGGATTCAAGAGGCAGGTTCTTGATCATTAACCTAGTCTCCTCCCCCAACTCCTC
N13	Adjacent tissue	chr16	−	6	4565	RBFOX1	RNA binding protein, fox-1 homolog (C. elegans) 1	+	6075152	TATGTCTTAAACATTATGGGAAAATTGTTAGGTGTTTTTGTATGCCTACAGCCTCCTAGTACAAAGATCTTTAATCTAATCTACTCCC
N104	Adjacent tissue	chr16	+	3	358	RBFOX1	RNA binding protein, fox-1 homolog (C. elegans) 1	+	7464068	TGTAACATGTACAGATTCTTATCTCACTCACCAATTTATGCCTACAGCCTCCTAGTACAAAGACCTTTAACCTAACCTCTTCCCCCAA
C109	Cancer	chr5	+	570	20953	SMAD5	SMAD family member 5	+	135491847	GAATGTGAACTACCTATAAAGAAGGCTATGTGCAGAGGTGAAGCGAAGTCGAAGAGAT
N109	Adjacent tissue	chr5	+	31	310	SMAD5	SMAD family member 5	+	135491847	TTGAAGAAGCTGTCATGAATGTGAACTACCTATAAAGAAGGCTACGTGCAGAGGTGAAGCGAAGT
C110	Cancer	chr5	+	30	1124	SMAD5	SMAD family member 5	−	135491851	TCATGAATGTGAACTACCTATAAAGAAGGCTACGTGCAGAGGTGAAGCGAAGT
C18	Cancer tissue	chr5	−	382	46659	TERT	Telomerase reverse transcriptase	−	1295135	ATCGCGGGGGTGGCCGGGGCCAGGGCTTCCCACAAAGTTGCATGGTGCTGGTGAACAGACCAATTTATGCCTACAGCCTCCTAGTACA
C100	Cancer	chr5	−	86	2724	TERT	Telomerase reverse transcriptase	−	1295396	GTGTCTGTGCCCGCGAATCCACTGGGAGCCCGGCGAAAAAGGTGCATGGGGCTTGTGGACCGACCAATTTATGCCTACCGCCCACTAG
C116	Cancer tissue	chr5	−	63	3789	TERT	Telomerase reverse transcriptase	−	1295441	CGCAGCTGCTCCGGGCGGACCCGGGGGTCTGGTGCCTACAGCCTCCTAGTACAAAGACCTTTAGCCTAATC
C105	Cancer	chr5	−	230	2853	TERT	Telomerase reverse transcriptase	−	1295563	GCTGTGGGGTAACCCGAGGGAGGGGCCATGATAGGACAAAGATCATTAACCTAATCTCCTCCCCCGACTCCTCCCAGTCTTTAAACAC
C117	Cancer tissue	chr5	−	270	7445	TERT	Telomerase reverse transcriptase	−	1295715	TGAAGGGGAGGACGGAGGCGCGTAGACGCTGGTGGACAGACCAATTTATGCCTACAGCCTCCGAGCACAAA
N117	Adjacent tissue	chr5	−	5	259	TERT	Telomerase reverse transcriptase	−	1295715	GAATGCCGGACGTGAAGGGGAGGACGGAGGCGCGTAGACGCTGGTGAACAGACCAATTTATGCCTACAGCC
C124	Cancer tissue	chr5	−	392	47491	TERT	Telomerase reverse transcriptase	−	1298846	CCTGCCATGGGGTGAAATTCTTTCTTTTTGAAAATACACAGGCTTTGAAGGATGCCTCAAGTGAGGTCGCT
N124	Adjacent tissue	chr5	−	55	2941	TERT	Telomerase reverse transcriptase	−	1298846	TCCCCTGCCATGGGGTGAAATTCTTTCTTTTTGAAAACACACAGTCTTTGAAGTATGCCTCAAGGTCGGTC

Sung *et al*. completed whole genome sequencing for 81 HBV-positive HCCs and identified 6 recurrent genes (*TERT*, *MLL4*, *CCNE1*, *SENP5*, *ROCK1* and *FN1*) [14]. *FN1 and TERT* were found as recurrent in both our and Sung *et al.*'s studies. The *TERT* gene was also reported by Fujimoto as a recurrent targeted gene of HBV [16]. If we consider the matches of our genes to the previous identified HBV targeted genes as recurrent, there are additional 9 recurrent genes (Table 2).

**Figure 7:**
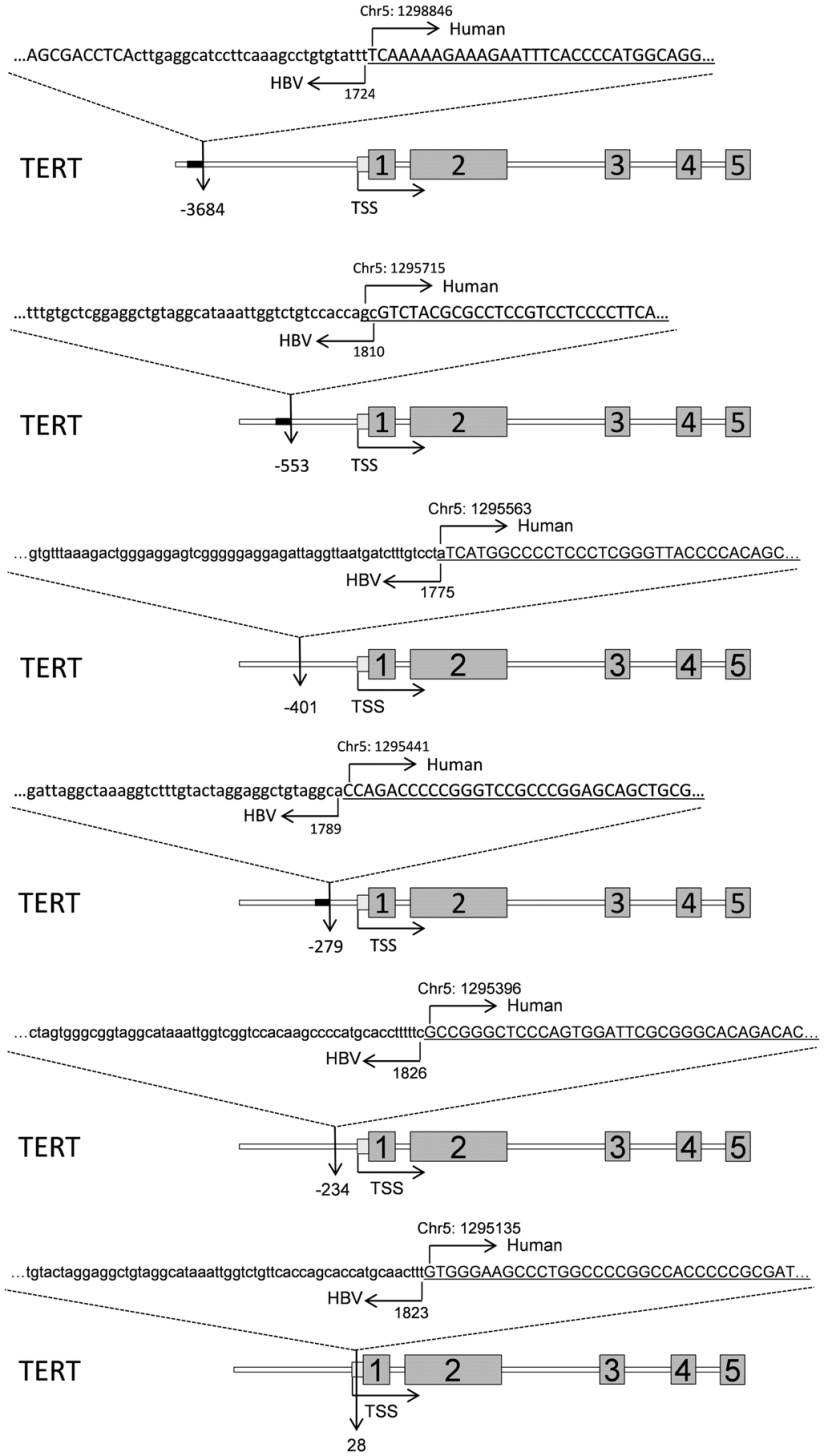
Illustration of the integrations targeted *TERT*. The sequences were representative Read 2 tags that cover the HBV integration sites to the *TERT* gene. HBV DNA was shown in lowercase, and the Human DNA was in uppercase and underlined. The numbered boxes represent nearby exons. The nucleotide position at the human and HBV were also indicated (based on GenBank AY800389.1 and Human hg19 assemble).

**Figure 8:**
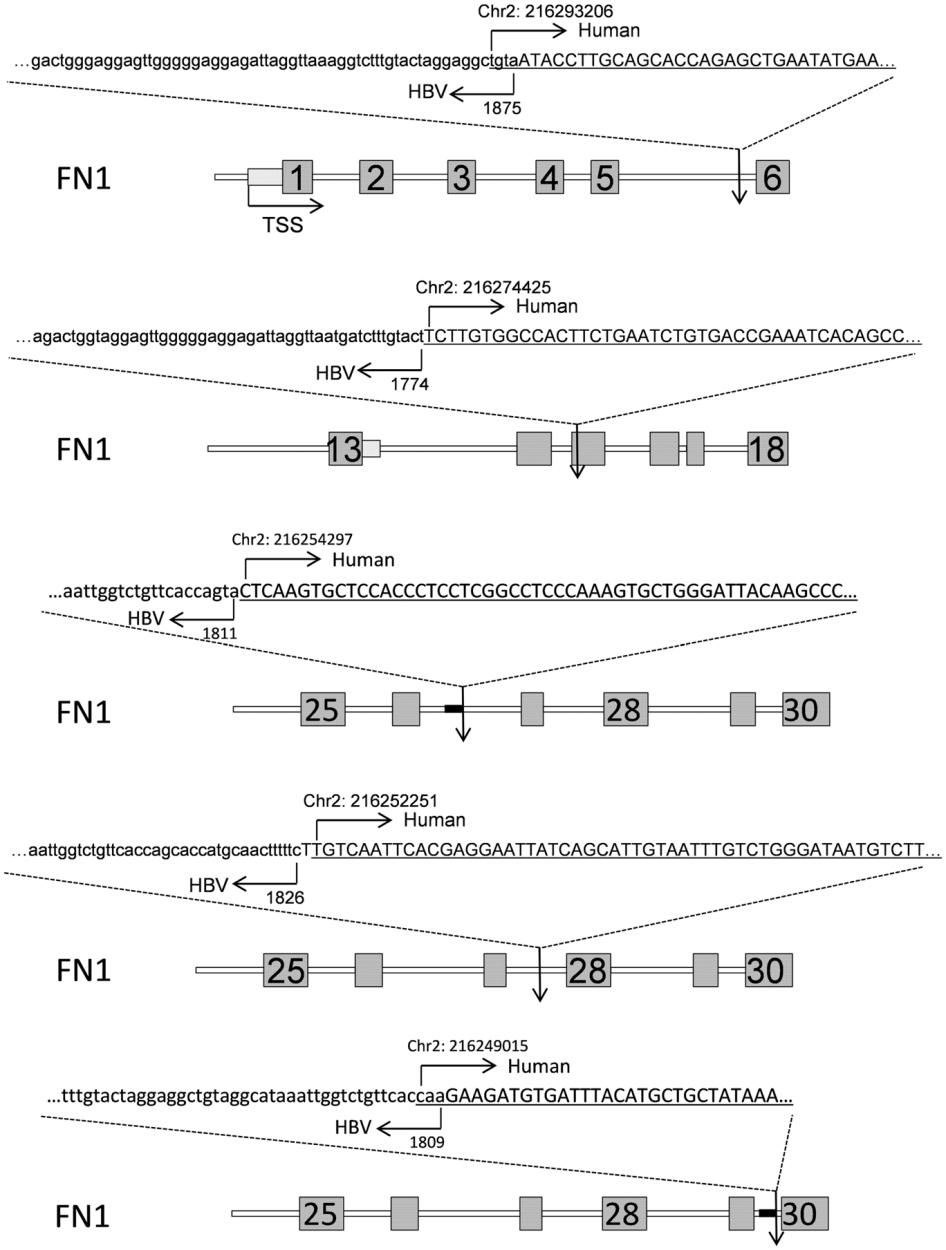
Illustration of the integrations in *FN1*. The sequences were representative Read 2 tags that cover the junction points of the HBV integration to the *FN1* gene. HBV DNA was shown in lowercase, and the Human DNA was in uppercase and underlined. The numbered boxes represent nearby exons. The nucleotide position at the human and HBV were also indicated (based on GenBank AY800389.1 and Human hg19 assemble).

To understand the consequences of HBV integration and the exact genomic features that is disrupted by the HBV integration, we created a BED format file to be used as custom track for UCSC genome browser to display the full list of HBV UIS with the chromosome integration junction (a 100 nucleotides were added to the junction position to create the ChromEnd column in the BED file) and with the total number of sequence reads covering the junction (Table S9). The positions of the recurrent integration sites in relationship to the gene structures were displayed using UCSC genome browser custom track (Figure S1). All 6 unique integrations targeting *TERT* inserted at the gene's promoter region. In particular, five HBV integrations in *TERT* located at the upstream the transcription start site, and one located in the 5′ UTR (Figure 7), similar to the HBV integrations target *TERT* identified in previous studies [9], [10]. Sung *et al.* identified 18 HBV integrations to intron 2 and 6, and to promoter regions of TERT [14], among which majority (15 of 18) of the integrations were in the promoter regions of TERT, similar to our observation. We also identified 5 unique HBV integrations into the FN1 gene. Four integrations were at the introns (at intron 5, 26, 27, 29) and a fifth integration into the exon 15 (Figure 8), which could either create a chimeric FN1-HBV fusion protein or could generate a truncated FN1 protein missing the exon 16–46. For the rest of HBV targets, the integrations were all at the introns of the genes except for an insertion into the exon of *PHACTR4* (Figure S1).

We performed RT-PCR on the available samples. For TERT gene, we compared one pair of samples where the only the cancer tissue harbors the integration, another pair of samples where both the HCC and the adjacent tissues harbor the HBV integration, and 6 pairs of samples where none of the tissues harbor the HBV integration. We found that expression ratios of TERT gene in HCC tissues to the tumor-adjacent tissues were 18.7, 1.0, and 0.33 (median of 6) respectively (Figure S2A). This is consistent with Sung *et al.*'s data showing HBV integration increase the expression of TERT gene [14]. This may due to the fact that HBV integrations to the TERT gene that we identified were all to the promoter regions.

We also conducted real time RT-PCR of the FN1 genes in three pairs of samples that were available. In two pairs of samples where FN1 integration was found only in the adjacent tissues, one showed no significant change (1.5 fold only), and the other showed reduction in the adjacent tissues harboring FN1 integration (Figure S2B). For the third pair of samples where FN1 integration was found in the cancer tissue only, the expression of FN1 was increased in the cancer tissue (Figure S2B). This might suggest that, for FN1 integration event, the effects on the FN1 might be position dependent or context (tumor vs. adjacent normal) dependent. The expression ratios of FN1 in the cancer to adjacent tissues in samples without HBV integration also varied greatly (Figure S2B, right panel). There was no significant association between the HBV integration in the FN1 gene and the expression changes (overall T-test P = 0.506 comparing samples with or without HBV integration, regardless of the HBV integrations were in the adjacent or cancer tissues) (Figure S2B, right panel). This is consistent with Sung *et al.*'s observation that there was no statistically significant difference for the FN1 expression between samples with or without HBV integration (P = 0.42), as shown in their figure 4b [14].

We also conducted RT-PCR with the available pairs of samples for other recurrent HBV targeted gene including ARHGEF12, CYP2C8, PHACTR4, PLXNA4, and RBFOX1. For ARHGEF12, the integration was found in the tumor-adjacent tissues in two pairs of available samples, and both integrations increased the expression of ARHGEF12 in the tumor-adjacent samples (7.4 and 1.8 fold respectively, tumor-adjacent to tumor ratios), which is statistically significant different from ARHGEF12 expression in 9 pairs of samples without ARHGEF12 integration (the average expression ratio of normal to tumor tissues was 1.1, T-test P = 0.04) (Figure S2C).

RT-PCR analysis of two HBV integrations into the PHACTR4 gene in the tumor-adjacent tissues revealed that the integration increased the expression 35.8 fold in one case, but did not increase the expression in another case (only 1.44 fold), compared with the matched cancer tissues that did not harbor the integration (data not shown). RT-PCR analysis of HBV integration to the PLXNA4 and CYP2C8 did not reveal significant changes in gene expression (data not shown).

For the RBFOX1 gene, HBV integration events reduced the expression of RBFOX1 in the tumor-adjacent tissues comparing to the cancer tissue in two match pairs of samples. In additional 6 pairs of samples without HBV integration in RBFOX1 that we analyzed, RBFOX1 gene were over expressed in 5 of the 6 pairs, but was greatly under expressed in one of the 6 pairs (maybe an outlier) (Figure S2D). Thus, the overall P value is not significant (P = 0.9).

Considering that a HBV target identified both in our study and the whole genome sequencing analyses of HCCs [14]–[16] is a recurrent event, we conducted an integrative analysis and identified a total of 20 recurrent HBV targeted genes (Table 4). Among them, 15 genes were either first identified in our studies or derived from the integrative analysis of our data with the recently published whole genome sequencing analysis.

**Table 4:** Recurrent targeted combining the integrative analysis.

Gene Symbol	Studies identified	Descriptions
ARHGEF12	our study	Rho guanine nucleotide exchange factor (GEF) 12
CYP2C8	our study	Cytochrome P450, family 2, subfamily C, polypeptide 8
PHACTR4	our study	Phosphatase and actin regulator 4
PLXNA4	our study	Plexin A4
RBFOX1	our study	
SMAD5	our study	SMAD family member 5
ADH1B	integrated analysis*	Alcohol dehydrogenase 1B (class I), beta polypeptide
CPS1	integrated analysis	Carbamoyl-phosphate synthetase 1, mitochondrial
ESRRG	integrated analysis	Estrogen-related receptor gamma
LRFN2	integrated analysis	Leucine rich repeat and fibronectin type III domain containing 2
MYOM1	integrated analysis	Myomesin 1, 185 kDa
RAI1	integrated analysis	Retinoic acid induced 1
CTDSPL2	integrated analysis	CTD (carboxy-terminal domain, RNA polymerase II, polypeptide A) small phosphatase like 2
LRP1B	integrated analysis	Low density lipoprotein-related protein 1B (deleted in tumors)
FN1	our study, Sung *et al.*, Jiang *et al.*	Fibronectin 1
TERT	our study, Sung *et al.*	Telomerase reverse transcriptase
MLL4	Sung *et al.*, integrated analysis	Myeloid/lymphoid or mixed-lineage leukemia 4
CCNE1	Sung *et al.*	Cyclin E1
SENP5	Sung *et al.*	SUMO1/sentrin specific peptidase 5
ROCK1	Sung *et al.*	Rho-associated, coiled-coil containing protein kinase 1

* Integrated analysis of our HBV target list with the recently published whole genome sequencing studies.

## Discussion

PCR-based methods including Alu-PCR [23], LM-PCR [24] and RS-PCR [10] were employed for isolating HBV integrants, but with biased amplification and limited sequencing capacity. Our massive anchored parallel sequencing (MAPS) approach avoided restriction bias by random fragmentation and takes full advantage of NGS with dual-functional adapters for comprehensive profile of HBV DNA integrations. Our approach is complementary to or an alternative approach to recent reports of applications of whole genome sequencing in the identification of HBV integration events [14]–[16].

We identified 286 UISs from 40 pairs of HBV-related HCC and tumor-adjacent tissues (Table S4), averaging at about 7 HBV integrations per individual. Recently, Sung *et al.* conducted whole genome sequencing at more than 30 fold coverage for 81 HBV positive HCCs and identified 399 HBV integration events using a criteria of more than 2 read pairs with close mapping positions linking an end of hg19 to an end of HBV, which resulted in the identification of an average of 4.9 HBV integration events per individual patient [14]. In another recently publication, Jiang *et al.* sequenced four HCC patients (only three are HBV positives) at 80X coverage and identified 255 HBV integration sites, which would generate at about 85 HBV integrations per HBV positive HCC patient [15]. Comparing the number of HBV integration per individual, our findings that the 7 HBV integrations per individual in our analysis is similar to the 4.9 HBV integrations per individual in Sung *et al.*'s study [14]. However, this might not be surprising as we sequenced two tissues (HCC and adjacent tissues) per individual while Sung *et al.* [14] only sequenced one tissue sample per individual. We found that adjacent tissues also harbor HBV integration events. However, the average numbers of HBV integration in our study and in Sung *et al.*'s analysis (about 7 and 5 respectively) are far off from the average number of HBV integrations (about 85) in Jiang *et al.*'s study. It is worth noting that, in the same issue of Nature Genetics, Fujimoto *et al.* found 23 HBV breakpoints using a criteria of 3 or more supporting read-pairs from 11 hepatitis B virus (HBV)-related HCCs with about 40X coverage whole genome sequencing, averaging at only about 2 HBV breakpoints per sample [16]. Further investigation would be necessary to resolve the discrepancy. There might be many potential reasons: first, there might be great HCC heterogeneities. Different subclasses of HCC, not yet defined, could have been used in the studies. Secondly, different HBV strains or sub-genotypes might have different capability of integration. Future analysis of the correlation between HBV subtypes and their integration frequencies would be necessary to address the question. Thirdly, different technologies might have different false positive and false negative HBV integration identification rates. The Complete Genomics' technology was used in Jiang *et al.*'s study [15] but the Illumina's platform was used in Sung *et al.*'s study [14]. The false positives might be derived from different methods of library construction and ligation procedures (which might generate chimeric ligation events). The false negative rate might be related to the sequence depth, sequence quality, the mapping parameters for the sequence reads and so on. To control the false positive identification, we made the efforts to use two controls: one negative genomic DNAs (without HBV integration) and another with a mixture of free HBV DNA and human genomic DNAs. The latter would control for chimeric ligation events. Using our analysis pipeline and criteria, we did not find any HBV integration events in either of the controls. Fourthly, the criteria to call an HBV integration event might be another factor affecting the number of HBV events called. Sung *et al.* and Jiang *et al.* called an HBV integration events if there are supported by at least two paired-end reads [14] or two chimeric reads [15]. Fujimoto *et al.* [16] and we used the criteria of at least 3 mapped read pairs.

We found more (about 6 times) HBV integration events in adjacent tissues (254 of 296 events, about 86%) than those in cancer tissues (42 of 296, about 14%). The median number of UISs in cancers was 1, whereas it is much higher at 7 in our tumor-adjacent tissues, consistent with previous reports showing nearly one insertion per HCC nodule [3], [25] and several (less than 10) integrations in chronic hepatitis patients [22], [26]. However, our observation is different from Sung *et al.*'s study where they found that HBV integration is observed more frequently in the tumors (86.4%) than in adjacent liver tissues (30.7%) [14]. We do not yet know the reason of this difference. We postulate that definition of adjacent tissues might be different in the two studies. In our study, the tumor-adjacent tissues might be closer to the cancerous tissues than the tumor-adjacent tissues in Sung *et al.*'s study [14]. In other word, one could postulate that the tumor-adjacent tissues closer to the cancer tissues might already contain precursor lesions while the tumor-adjacent tissues further away might be normal liver tissues. Therefore, we have adopted the term ‘tumor-adjacent’ tissue instead of the term normal tissue or the ‘adjacent non-tumor’ tissue in this manuscript.

In addition to the lower number of UISs in cancer tissues described above, the HBV insertional frequencies in cancer tissues were statistically larger than that in cancer-adjacent tissues (p<0.0001), which was also observed by Jiang *et al.* in their study [15]. Taking together, this might suggest a higher level of clonal expansion of cancer hepatocytes in liver cancer tissues compared to cancer-adjacent tissues, which was also suggested by Jiang *et al.*'s whole genome sequencing of 4 hepatocellular carcinoma patients [15]. As suggested by a previous study [3], it seems that viral integration occurred during HBV chronic infection before HCC initiation. Hepatocytes with HBV integration underwent certain rounds of expansion during chronic hepatitis; some of the cells participated in the formation of so called focal proliferative lesions and gained growth advantages, resulting in clonal expansions during tumorigenesis.

We also compared our list with the list of common CISs for HCC-associated genes identified in a transposon-based (*Sleeping Beauty*) insertional mutagenesis screening in mice [27]. There are no common genes between the lists. However, two genes in the mouse CISs belong to the same family members with two genes in our list: *zbtb20* in mouse vs. ZBTB16 in human, and *slc25a13* in mouse vs. SLC2A13 in human. This prompted us to analyze the two lists to see if they have common pathways. Using the DAVID program (david.abcc.ncifcrf.gov/) to analyze the pathways enriched in both lists, we found that they shared three enriched (P<0.05) pathways, which are the renal cell carcinoma pathway, focal adhesion pathway, and the ErbB signaling pathway (data not shown).

Once, integrations of virus into host genomic DNA was thought to be totally random [28]. More recent studies, however, suggested that integration of various viruses into human genome has distinct preferences [29]–[31]. The profile of HBV insertion sites in this study also demonstrated that HBV integration preferentially landed in transcription units and specific chromosomes. Using a randomly generated *in silico* integration site dataset, we demonstrated that viral integration by HBV favored transcription units (*P*<0.0001) and chromosome 17. Preference for HBV integration to chromosomes 11 and 17 has been reported in a previous study [4] and a preference for chromosome 3 has been reported in chronic hepatitis tissues without HCC by Alu-PCR [26].

A possible oncogenic contribution of HBV DNA integration involves the insertion of viral DNA into cellular genomic regulatory regions or coding regions, resulting in modification of gene expression (cis-activation) or production of structurally and functionally aberrant cellular or hybrid proteins. Previously identified targeted genes include the *myc* family oncogenes [32], [33], *CCNA2* [8], [34], [35], the *RARB* [7], [36], the mevalonate kinase (*MK*) [37], [38], the carboxypeptidase N (*CPN2*) [39], sarco/endoplasmic reticulum ralcium ATPase (*SERCA*) [40] and *TERT* [9]. Whether any oncogenic proteins such as chimeric HBV-host gene fusion proteins would be generated from the 286 UISs remains to be investigated. We found that 8 target genes were inserted by HBV for more than once (Table 3), including *ARHGEF12*, *CYP2C8*, *FN1*, *PHACTR4*, *RBFOX1*, *SMAD5* and *TERT*. Previously, *TERT* and *MLL4* (myeloid/lymphoid or mixed-lineage leukemia 4) were identified as recurrent HBV targeted genes [10], [16], [41]. Here we identified additional 8 genes as recurrent target of HBV integration, expanding the list of human host genes targeted by recurrent HBV integration. Further additional 9 recurrent genes were identified if we consider the matches of our HBV targeted genes to the previously identified HBV targeted genes [14], [15], expanding the novel recurrent target genes to 14 (Table 4, identified under our study or integrated analysis).

Fibronectin 1 is a high-molecular weight (∼440 kDa) glycoprotein of the extracellular matrix and contain binding domains for binding to cell surface integrins and other extracellular matrix components such as collagen, fibrin, actin and heparan sulfate proteoglycans (e.g. syndecans), and it plays a major role in cell adhesion, motility, opsonization, as well as in wound healing and embryonic development [42]. Altered fibronectin expression, degradation, and organization have been implicated in cancer and fibrosis [43]. We identified five HBV integration sites in the *FN1* genes: four integrations into the intron 5, 26, 27 and 29, and the fifth into the exon 15 (Figure 8). Whether these HBV integrations could alter *FN1* expression or had any causative role in HCC carcinogenesis remains to be determined. HBV integration into *FN1* exon 15 could either create a chimeric FN1-HBV fusion protein or could generate a truncated FN1 protein missing the exon 16–46, which would shorten the transcript from 8449 nucleotides (NM 002026) to less than 2565 nucleotide (exon 15 ends at nucleotide 2565). The protein product would shorten from 2355 AA to less than 766 AA, which would reduce the number of fibronectin type-III repeats from 16 in FN1 to about 1.5 in the truncated version. Furthermore, the truncated FN1 would miss the heparin-binding and syndecan-binding domains. Sung *et al.* recently also reported 5 HBV integrations into the *FN1* gene; however, all are to the introns [14].

Using PCR, we were able to confirm the HBV genomic integration into FN1 using the genomic DNAs used for MAPS library preparation and sequencing. However, when we attempted to take a second section of the tissue to prepare RNA and to perform RT-PCR to identify chimeric transcripts that could be potentially generated from the integration, we were not able to identify chimeric HBV-FN1 RNA transcripts. It could be possible that integration prevented transcription or due to different section of the tissues were used as tumors usually show great heterogeneity. Further experimentation would be necessary to figure out the consequence of the HBV integration into the FN1 region.

Considering that 7 genes (*PTPRJ, CNTN6, IL12B, MYOM1, FNDC3B, LRFN2, FN1*)) contain the IPR003961: Fibronectin, type III domain (Table S6), we speculate that fibronectin type III repeat or like fibronectin type III repeat fold domain might play a role in HCC. Zhang *et al.* recently showed that up-regulated miR-143 transcribed by nuclear factor kappa B (*NFKB*) promotes the invasion of HBV-HCC by repressing expression of fibronectin type III domain containing 3B (*FNDC3B*) [44]. Further investigation is warranted. However, we believe that would be the task for a future paper, as it will take considerable effort and time to investigate the mechanism involved. When Tomlins *et al.* first identified recurrent fusion of TMPRSS2 and ETS transcription factor genes in prostate cancer in a science publication [45], it took the field several years to figure out the mechanism involved [46].

We used quantitative RT-PCR to assess the effects of HBV integration on the targeted gene expression in available matched pairs of tumor-adjacent and tumor samples. We found that HBV integration into the TERT gene increases its expression (Figure S2A). This is consistent with Sung *et al.*'s data showing HBV integration increase the expression of TERT gene [14], and might also reflect the fact that HBV integrations to the TERT gene that we identified were all in the promoter regions. In addition, we also found that HBV integration to the ARHGEF12 gene in the tumor-adjacent tissues increased their expression (Figure S2C). However, for the rest of recurrent targeted genes, the effects on the expression are more complicated: they either showed no effects or had sample-specific effects. Our observation on the effect of HBV integration to its targeted genes is similar to what Sung *et al.* observed, where they found that three of the six HBV targeted genes showed changed expression level, but another three of the six targeted genes showed no statistically significant changes in gene expression (Figure 4b in [14]).

To determine the sensitivity of our MAPS approach, we tested to see if we can identify the same HBV integrations in data from replicate runs generated during our optimization of the protocol. The two replicate experiments were performed using the same pool of DNAs of 6 pairs of samples plus two controls, but differ slightly in the modifications of primers used: the first was with the single 5 nt barcode and the second with the paired barcodes. For the first replicate, we identified 54 HBV integration events, and for the second replicate, we identified 68 HBV integration events. 40 HBV integration events are in common (data not shown). We identified more HBV integration events for the second data sets as we had a deeper sequencing coverage (a total of 30,761,630 reads vs. 24,886,345 in the first dataset). A total of 82 HBV integration events were identified combining both replicates. 14 were uniquely identified in the first replicate. Therefore, it is possible that we might miss 14 of 82 (17%) of the HBV integration events if we only sequenced 30 million per 14 samples (i.e. a sequence depth of about 2.2 million reads per sample). In a random sampling analysis (Figure 5D), we showed that indeed the curve for the number of HBV integration events identified per million of additional sequence reads starts to become flat at 10 million reads (for 14 samples, this would correspond to an average at about 700 K per sample), but it continues to rise, although slowly. As the average sequence depth is about 2.2–2.5 million reads per sample in our analysis, we would miss at least 17% of the HBV integration events. It is worth to point out that our sensitivity is not 100%, but our specificity is high as we controlled for the false positive identification using two controls: one negative genomic DNAs (without HBV integration) and another with a mixture of free HBV DNA and human genomic DNAs. The latter would control for chimeric ligation events. Using our analysis pipeline and criteria, we did not find any HBV integration events in either of the controls.

There are 47 HBV integration events that could not be annotated (Table S4). We searched them against the human repeat sequence using the RepeatMasker program (www.repeatmasker.org). Seven of the integrations were found in the repeat regions including LINE, LTR/ERV1 and SINE/Alu repeats (Table S11).

In summary, this study highlights these novel findings: 1) higher number of HBV integration events were found in cancer adjacent tissues than in HCC tissues, suggesting a clonal expansion process during HCC development; 2) fibronectin and its associated genes including fibronectin type III-like fold domain containing genes are frequently targeted by HBV DNA integration, and 3) 14 novel recurrent HBV targeted genes were identified when combined our list with recently published HBV targeted gene lists [14]–[16], greatly expanding the recurrent HBV human genome integration gene list. This study, together with recent global survey of HBV integration events [14]–[16] provides a good foundation for further experimentation and understanding the mechanism of HBV-related HCC.

## Materials and Methods

### Ethics statement

Informed consent was obtained from each patient and the ethics committee of the First Affiliated Hospital, College of Medicine, and Zhejiang University approved all aspects of the study.

### Isolation and identification of HBV DNA integrations

The study population comprised 40 HBV-positive HCCs (Table S10).

Two micrograms of the genomic DNA in a volume of 60 µl of Elution Buffer (EB, Qiagen) were fragmented by Bioruptor (Diagenode Inc.). The operating conditions were the following: ice-water batch refreshed every 5 minutes of sonication, intensity level H, 5 sec power on and 5 sec power off, a total of 25–35 minutes of sonication depends on the results of gel electrophoresis. Sonication resulted in the sizes of DNA fragments ranging from 1000 bp to 100 bp. DNA ends were then end-repaired using 15 units of T4 DNA polymerase (New England Biolabs, NEB), 5 units of DNA polymerase I Klenow fragment (NEB), 50 units of T4 polynucleotide kinase (NEB) and 0.8 mM of dNTP (NEB) in T4 DNA ligase buffer (NEB) at 20°C during 30 min. DNA fragments was then purified using a Qiaquick PCR purification kit (Qiagen) and eluted in 30 µl of EB. Addition of an adenosine at the 3′ ends of the DNA was performed by adding 0.2 mM of dATP (TaKaRa) and 15 units of Klenow Fragment 3′ to 5′ exo- (NEB) in NEB2 buffer (NEB) at 37°C for 30 min. DNA was then purified using a Qiaquick PCR purification kit and eluted in 30 µl of EB. Twenty-six different linkers were constructed, each one with a specific 6 bp tag (Table S2) to mark each DNA sample before amplification.

Two rounds of PCR were performed to amplify target sequences (integrants) using KOD-Plus-Neo DNA polymerase (Toyobo Co., Ltd.). A first round of PCR was performed with the HBX1 primer and the walking primer PE 2.1 for 25 cycles. Target sequences were amplified in 50 µl reaction volume containing 1X PCR Buffer for KOD-Plus-Neo DNA polymerase, 1.5 mM MgSO4, 200 µM each of dATP, dCTP, dGTP and dTTP, 300 nM each of the HBX primer and the walking primer, 500 ng of the walking library and 0.02 units/µl KOD-Plus-Neo DNA polymerase. Amplification condition comprised of a 2 minutes activation step at 94°C followed by 25 cycles of 95°C for 15 seconds, and 68°C for 1 min. A second round of PCR was performed with the PE1-Barcode-HBx2 primer and the walking primer PE 2.2. One micro liter of the first round PCR product was used as the template in a 50 µl reaction. All components were at the same concentrations as the first round PCR, and cycling conditions were the same as well. Reactions of nested PCR were diluted 10 fold in sterile distilled water.

Five microliters of the diluted product were used to enrich target templates while introducing the full-length Illumina PE sequencing adapters. Enriched libraries were then purified by gel electrophoresis separately. For each sample, a gel slice containing the DNA material in around 500 bp was excised with clean scalpels. Libraries were purified with the QIAquick Gel Extraction Kit (Qiagen Inc.), and then the 12 barcoded samples were pooled together with two control libraries at equimolar ratios. These two negative control libraries were used to help evaluate this method. HBV-free genomic DNAs isolated from the glioblastoma cell line U251, either mixed with HBV Genomic DNA or alone, were subjected to the MASP approach described above. Pooled PE sequencing libraries were quantified with Qubit (Invitrogen Inc.). 95×95 or 78×78 cycles of PE sequencing reactions were performed on an Illumina GA II according the standard protocol.

### Double barcodes for multiplex analysis

Six or sixteen pairs (cancer and adjacent tissues) of HBV-positive hepatocellular carcinomas (HCCs) were analyzed in each multiplex lane using DNA bar coding, a 5 nt barcode on Read 1 and a 6 nt barcode on Read 2. The barcoded primers were listed in Table S2 and S3.

Illumina image analysis algorithms use the first 4 or 5 cycles of pictured images to identify the positions of individual cluster (11). In MAPS, Read 1 ends sequences extended from specific nest primers (HBX secondary primer) are of low-diversity. If there is no barcode bases added before the primer, the position determining base signals on the image tend to be severely skewed (data not shown), which compromises much of the sequencing capacity. As a result, we used the 5 nt barcode in PE1-Barcode-HBX2 primers to analyze pooled DNA samples.

Multiple parallel nested PCR reactions provide an ideal scenario for migration of amplicons between samples. Barcoded HBX primers crosstalk with integrant templates from other samples, and the crosstalk products were simultaneously sequenced. In this study, however, insertions were assigned to original samples by comparing their frequencies in each sample (data not shown). To secure sequence assignment and suppress PCR contaminations, we additionally introduced the 6 nt barcode to Read 2. Using the paired barcodes, we eliminated potential contaminations and assign insertions to their samples with confidence.

### Sequencing data analysis and insertion identification

Images deconvolution and quality values calculation were performed using the Goat module of the Illumina pipeline. Read 1 sequences of Illumina PE sequencing were assigned to individuals according to the 5 nt barcodes using SplitFastQIndex.py from http://bioinf.eva.mpg.de/multiplex/. HBV containing sequences were filtered from non-HBV sequences using SOAP (Short Oligonucleotide Analysis Package) (http://soap.genomics.org.cn/) matching with all HBV genomes references we downloaded from NCBI. The corresponding Read 2 sequences with the correct 6 nt barcode of each sample were picked out using the SplitFastQIndex.py script. The selected Read 2 sequences were then aligned with HBV genomes to filter out HBV derived sequences by SOAP. The remaining sequences were aligned with Human Genome to map potential insertions.

HBV containing sequences from Read 1 were identified by aligning them to all HBV genome references from the GenBank database isolated in China. The corresponding Read 2 was aligned to the HBV genomes to filter out HBV derived sequences. The remaining Read 2 was aligned to the human genome (build hg19) to map their locations for putative insertion site identification.

We calculated the frequency and coverage of the insertion sites. Read 2 sequences were viewed as redundant copies of unique tags, if they mapped the same coordinates of the Human Genome. The coverage is the average copy numbers of all the unique tags. To be conservative, we considered different tags offset by less than 2 nucleotides at the ends as one effective unique tag. We also set the criteria that a putative integration should have at least three effective unique tags. Based on the sequencing data from the negative controls, we selected candidate integration site when there is more than 10 fold of overall coverage, and 2/3 of the unique tags have the coverage over 3 have 10 fold of overall coverage. We viewed those putative integrations as authentic if there is Read 2 sequence covering the integration junction point.

### Validation by nested PCR assays

The first round of the PCR was performed with the HBX1 primer and a site-specific first round primer for 25 cycles, with 50 ng of the genomic DNA as templates. The 20 µl reaction contained 1X PCR Buffer for KOD-Plus-Neo DNA polymerase, 1.5 mM MgSO4, 200 µM each of dATP, dCTP, dGTP and dTTP, 200 nM each of HBX primer and site-specific primer, and 0.02 units/µl KOD-Plus-Neo DNA polymerase. Thermal conditions were consisted of a 2 minutes activation step at 94°C followed by 25 cycles of 95°C for 15 seconds, and 68°C for 1 min. 1 µl of the products were diluted by adding 49 µl sterile distilled water to each. 0.5 µl of the dilution was subjected to the second round, 30 cycles of 10 µl reactions with the nest site-specific primer and HBX 2 primer (ACTTCGCTTCACCTCTGCACGT). Concentrations and thermal condition were similar to the first round; except that extension time at 68°C was reduced to 45 sec. Final products were visualized on 2% gels.

### Semi-quantitative analysis of UIS abundance

The relative abundance of a given UIS was calculated from the number of amplicons of different length (i.e. different shear site). Semi-quantitative PCR was performed to evaluate the quantification within a single sample and among multiplex samples. Duplicate experiments were compared to examine the consistency of the quantification. To evaluate the sequencing depth for quantitative analysis, we also did a rarefaction analysis of raw sequence data using a random sampling approach.

### Generation of random dataset

To determine if there were favored chromosomes for HBV integration *in vivo*, we generated a comparative random set of 8,015 genomic positions *in silico*. A set of 10,000 random numbers was generated by Perl script, between 1 and 3,095,677,412, the size of the human genome. These random numbers were then converted into chromosomal positions. We retrieved 35 nt sequences that ended with these positions. We then mapped those sequences to the Human Genome using SOAP, resulting in 8,015 unique matches.

### Rarefaction analysis

We randomized the total sequence reads 10 times into 39 size bins to evaluate saturation of the insertion pool by current MAPS strategy. As the total reads was 30,761,630, the largest size of randomized sets was set to 30 million (M). The first 9 smallest sizes of subset range from 0.1 M to 0.9 M, at an interval of 0.1M. The other 30 subsets range from 1 M to 30 M, at an interval of 1M. We determined the numbers of putative integrations in all the randomized subsets according to the criteria above. The results were plotted using Sigma Plot (version 11.0; SPSS Inc.). Regression analysis was also performed to fit the rarefaction curve into the double rectangular hyperbola curve model.

### Mapping and functional annotation of UIS

RefSeq genes or its vicinity (150 kb upstream of the transcript start sites and 150 kb downstream of the transcript stop sites) inserted by HBV DNA were determined by home-written Perl scripts. The nearest RefSeq gene was selected for each integration site, and was referred as HBV integration target gens in the text. We used the Idiographica (http://www.ncrna.org/idiographica/) to generate a high-quality ideogram of human chromosomes with HBV integration sites. We performed Gene Ontology (GO) analysis using the GO miner program (http://discover.nci.nih.gov/gominer/index.jsp). Additional Functional annotation of the target genes tagged by integration was performed using DAVID (http://david.abcc.ncifcrf.gov/) Bioinformatics Resources. Categories of KEGG PATHWAY, and INTERPRO were included for further interpretation.

### Real-time PCR assay

Total RNAs were extracted using mirVana miRNA Isolation Kit (Ambion, Life Technologies) according to the manufactory's instruction on total RNA extraction. The concentration and quality of RNAs were determined by Nanodrop and gel electrophoresis. RT-PCRs were performed with M-MLV Reverse Transcriptase (Promega) using 2 µg RNAs, and real-time PCRs were performed using SYBR Premix Ex Taq II (TaKaRa) in 20 µl in duplicates.

### Statistical analysis

Statistical tests were performed using SAS 9.2. The Shapiro-Wilk test or the Kolmogorov-Smirnov test was used for determining normality of data. The Mann–Whitney test was performed to analyze the difference in UISs abundance and viral integration frequency between tumor and non-tumorous groups. Integration rate in RefSeq genes compared to random was tested using the Pearson's chi-squared test. A p-value <0.05 was considered as significant for all the tests.

## Supporting Information

Figure S1 Schematics showing the HBV UIS along with the genomics features of the five recurrent targeted genes from UCSC genome browser. Vertical bars above the Refseq gene structures including exon and introns showed the HBV UIS identified.
(TIF)

Figure S2 Influence of HBV integration on gene expression in matched pairs of HCC and tumor-adjacent tissues. Gene expression was normalized to GAPDH and represented as either the tumor/adjacent-tumor or the adjacent-tumor/tumor gene expression ratios. Standard Box-and-whisker plot schema was used with the bottom and the top of the box showing the 25^th^ and 75^th^ percentiles of the data, horizontal lines showing the means and the error bars showing 95% CI for mean. A. Box-and-whisker plots of gene expression of the TERT gene in matched pairs with HBV integration in the cancer tissue only, matched pairs with HBV integrations in both tissues, and matched pairs of samples without HBV integration to the TERT gene. B. Left panel, bar charts showing the expression ratios of FN1 in the tissues with HBV integration (tumor or tumor-adjacent) to the tissues without HBV integration (tumor or tumor-adjacent). Please note in two cases (sample 19 and 106), the HBV integrations were in the tumor-adjacent tissues, and in one case (sample 25), it was in the tumor tissue. Right panel, Box-and-whisker plots of gene expression of the FN1 gene in matched pairs with HBV integration in the cancer tissue only, matched pairs with HBV integrations in both tissues, and matched pairs of samples without HBV integration to the FN1 gene. C. Box-and-whisker plots of gene expression of the ARHGEF12 gene in matched pairs with HBV integration and matched pairs without HBV integration. D. Left panel, bar charts showing the expression ratios (tumor/adjacent-tumor) of RBFOX1 in individual samples with HBV integration or without HBV integration. Right panel, Box-and-whisker plots of gene expression of the RBFOX1 gene in matched pairs with HBV integration and matched pairs without HBV integration.
(TIF)

Table S1 Sequences of the MAPS primers.
(DOC)

Table S2 The 6 nt barcode sequences for PE 2 Walking Adapter.
(DOC)

Table S3 The 5 nt barcode sequences incorporated with nested primers.
(DOC)

Table S4 Summary of HBV targeted genes identified in HCC and adjacent tissues.
(XLS)

Table S5 PCR confirmation of putative HBV integration sites.
(XLS)

Table S6 Enriched GO terms for HBV targets identified.
(XLS)

Table S7 Enriched INTERPRO domains for HBV targets identified.
(XLS)

Table S8 Enriched KEGG pathways for HBV targets identified.
(XLS)

Table S9 Bed file for the HBV integration sites.
(TXT)

Table S10 Clinical information of the tissues used in the study.
(DOC)

Table S11 HBV integrations that map to the human repeat sequences.
(XLSX)
