# Supplementary figures and images for: Recurrent Targeted Genes of Hepatitis B Virus in the Liver Cancer Genomes Identified by a Next-Generation Sequencing–Based Approach
Source: PLoS Genet. 2012 Dec 6;8(12):e1003065. doi: 10.1371/journal.pgen.1003065 (PMC3516541; doi:10.1371/journal.pgen.1003065)

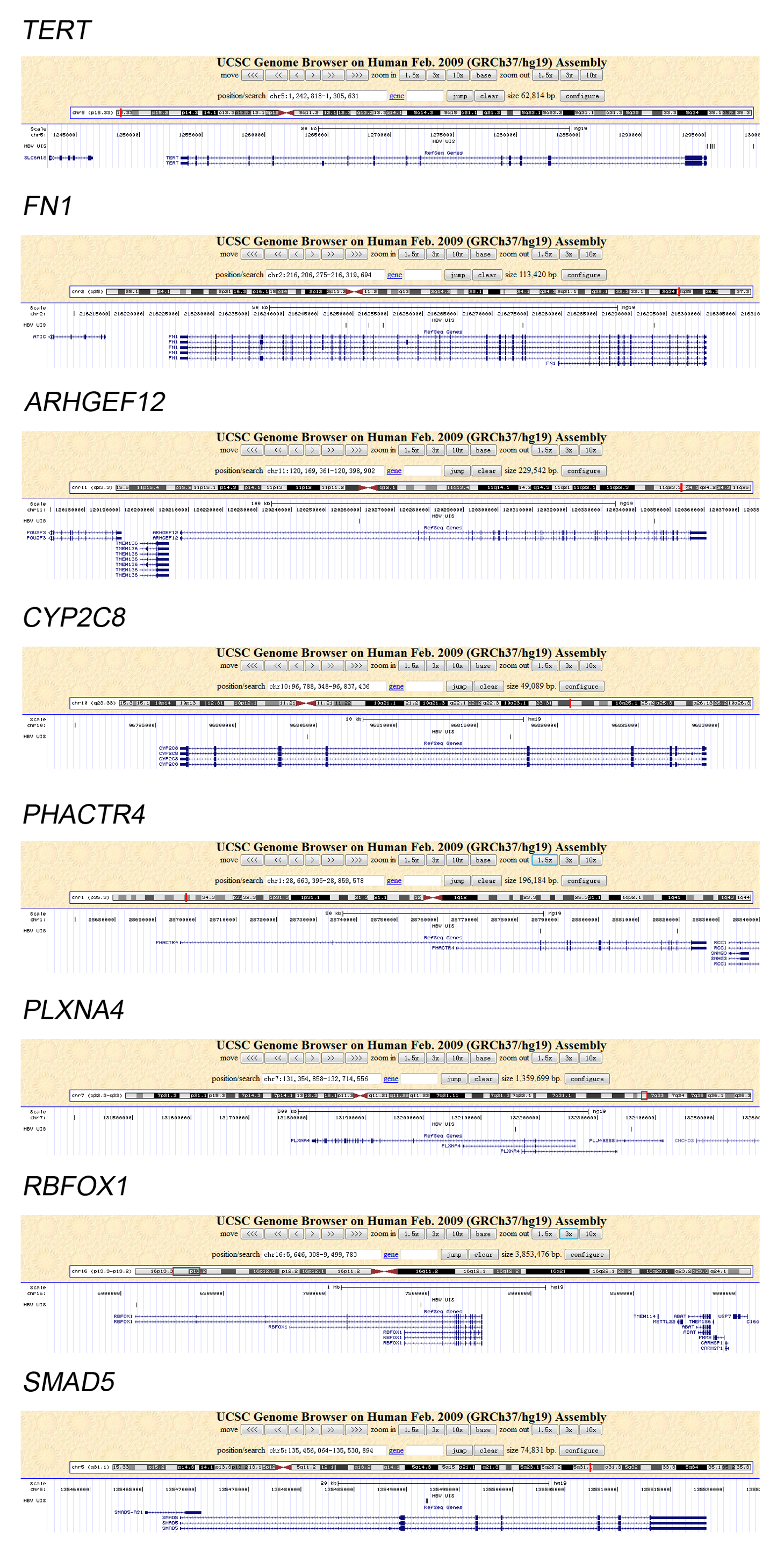

Supplement: Figure S1 — Schematics showing the HBV UIS along with the genomics features of the five recurrent targeted genes from UCSC genome browser. Vertical bars above the Refseq gene structures including exon and introns showed the HBV UIS identified. (TIF) [file pgen.1003065.s001.tif]

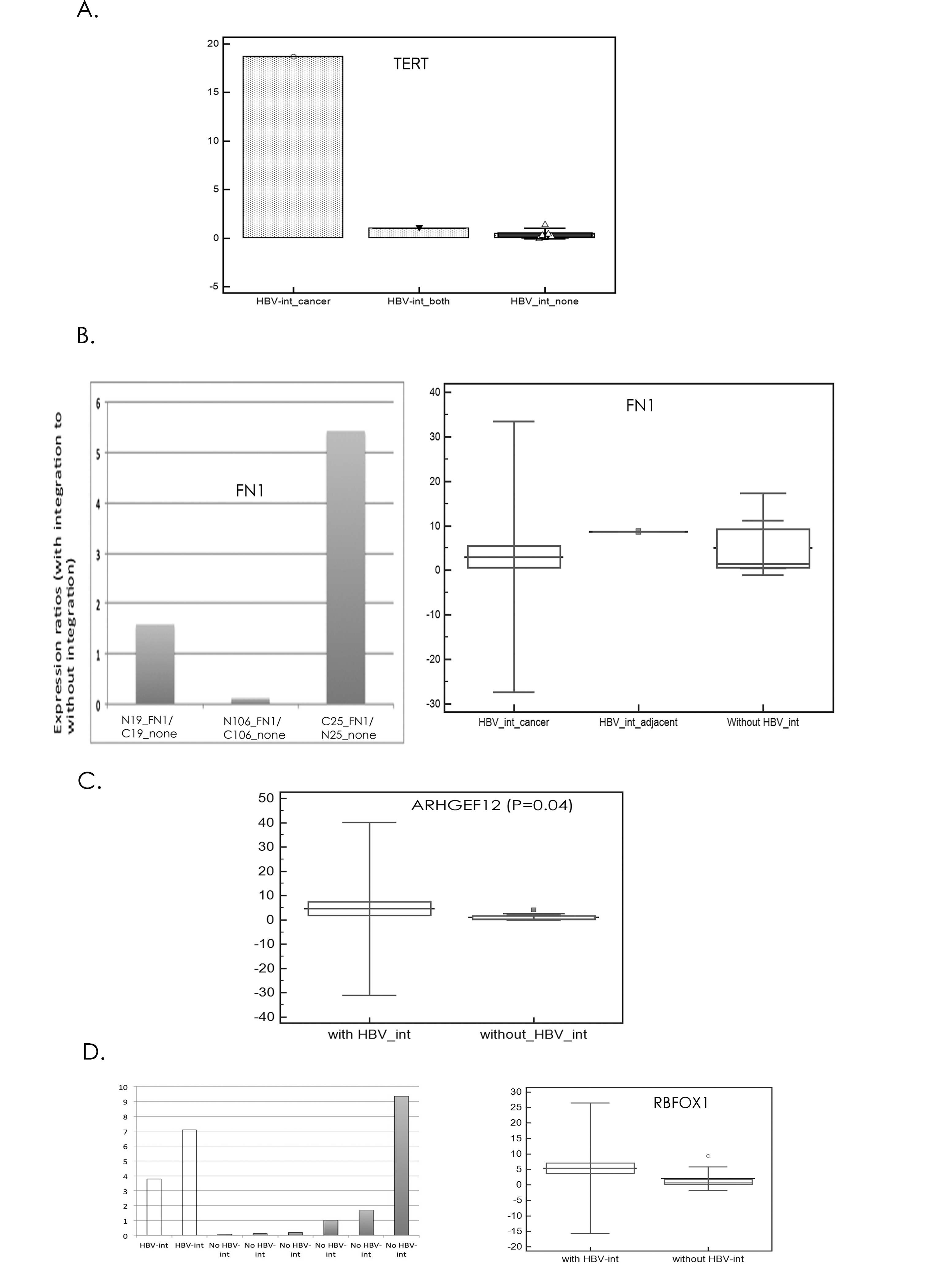

Supplement: Figure S2 — Influence of HBV integration on gene expression in matched pairs of HCC and tumor-adjacent tissues. Gene expression was normalized to GAPDH and represented as either the tumor/adjacent-tumor or the adjacent-tumor/tumor gene expression ratios. Standard Box-and-whisker plot schema was used with the bottom and the top of the box showing the 25th and 75th percentiles of the data, horizontal lines showing the means and the error bars showing 95% CI for mean. A. Box-and-whisker plots of gene expression of the TERT gene in matched pairs with HBV integration in the cancer tissue only, matched pairs with HBV integrations in both tissues, and matched pairs of samples without HBV integration to the TERT gene. B. Left panel, bar charts showing the expression ratios of FN1 in the tissues with HBV integration (tumor or tumor-adjacent) to the tissues without HBV integration (tumor or tumor-adjacent). Please note in two cases (sample 19 and 106), the HBV integrations were in the tumor-adjacent tissues, and in one case (sample 25), it was in the tumor tissue. Right panel, Box-and-whisker plots of gene expression of the FN1 gene in matched pairs with HBV integration in the cancer tissue only, matched pairs with HBV integrations in both tissues, and matched pairs of samples without HBV integration to the FN1 gene. C. Box-and-whisker plots of gene expression of the ARHGEF12 gene in matched pairs with HBV integration and matched pairs without HBV integration. D. Left panel, bar charts showing the expression ratios (tumor/adjacent-tumor) of RBFOX1 in individual samples with HBV integration or without HBV integration. Right panel, Box-and-whisker plots of gene expression of the RBFOX1 gene in matched pairs with HBV integration and matched pairs without HBV integration. (TIF) [file pgen.1003065.s002.tif]
