# Supplementary material for: Recurrent Targeted Genes of Hepatitis B Virus in the Liver Cancer Genomes Identified by a Next-Generation Sequencing–Based Approach
Source: PLoS Genet. 2012 Dec 6;8(12):e1003065. doi: 10.1371/journal.pgen.1003065 (PMC3516541; doi:10.1371/journal.pgen.1003065)
Supplement: Table S1 — Sequences of the MAPS primers. (DOC) [file pgen.1003065.s003.doc]

**Table S1: Sequences of the MAPS primers**

| **Name** | **Sequence (5’–3’)** |
| --- | --- |
| PE 2 Adapter | CAAGCAGAAGACGGCATACGAGATCGGTCTCGGCATTCCTGCTGAACCGCTCTTCCGATCTNNNNNN*T |
| Walking Adapter 2 | ^#^PO4-NNNNNNAGATCGGAAGAGCGAGCACATCCCTTTCTCACA |
| PE 2.1 | CAAGCAGAAGACGGCATACGAGATC |
| PE 2.2 | GGTCTCGGCATTCCTGCTGAACC |
| HBX1 | TCTCATCTGCCGGACCGTGT |
| PE1-Barcode-HBX2 | CACTCTTTCCCTACACGACGCTCTTCCGATCTNNNNNACTTCGCTTCACCTCTGCACGT |
| PE 1 | AATGATACGGCGACCACCGAGATCTACACTCTTTCCCTACACGACGCTCTTCCGATCT |
| PE 2 | CAAGCAGAAGACGGCATACGAGATCGGTCTCGGCATTCCTGCTGAACCGCTCTTCCGATCT |

*Phosphorothioate bond between the last two bases. ^#^ Walking Adapter 2 oligos were 5’ phosphorylated.
