# Supplementary material for: Recurrent Targeted Genes of Hepatitis B Virus in the Liver Cancer Genomes Identified by a Next-Generation Sequencing–Based Approach
Source: PLoS Genet. 2012 Dec 6;8(12):e1003065. doi: 10.1371/journal.pgen.1003065 (PMC3516541; doi:10.1371/journal.pgen.1003065)
Supplement: Table S2 — The 6 nt barcode sequences for PE 2 Walking Adapter. (DOC) [file pgen.1003065.s004.doc]

**Table S2: The 6 nt barcode sequences for PE 2 Walking Adapter**

| **Barcode tags** | **PE 2 Adapter sequences*** | **Walking Adapter 2 sequences#** |
| --- | --- | --- |
| ACATCG | CAAGCAGAAGACGGCATACGAGATCGGTCTCGGCATTCCTGCTGAACCGCTCTTCCGATCTACATCG (PS) T | PO4-CGATGTAGATCGGAAGAGCGAGCACATCCCTTTCTCACA |
| GCCTAA | CAAGCAGAAGACGGCATACGAGATCGGTCTCGGCATTCCTGCTGAACCGCTCTTCCGATCTGCCTAA (PS) T | PO4-TTAGGCAGATCGGAAGAGCGAGCACATCCCTTTCTCACA |
| TGGTCA | CAAGCAGAAGACGGCATACGAGATCGGTCTCGGCATTCCTGCTGAACCGCTCTTCCGATCTTGGTCA (PS) T | PO4-TGACCAAGATCGGAAGAGCGAGCACATCCCTTTCTCACA |
| CACTGT | CAAGCAGAAGACGGCATACGAGATCGGTCTCGGCATTCCTGCTGAACCGCTCTTCCGATCTCACTGT (PS) T | PO4-ACAGTGAGATCGGAAGAGCGAGCACATCCCTTTCTCACA |
| ATTGGC | CAAGCAGAAGACGGCATACGAGATCGGTCTCGGCATTCCTGCTGAACCGCTCTTCCGATCTATTGGC (PS) T | PO4-GCCAATAGATCGGAAGAGCGAGCACATCCCTTTCTCACA |
| GATCTG | CAAGCAGAAGACGGCATACGAGATCGGTCTCGGCATTCCTGCTGAACCGCTCTTCCGATCTGATCTG (PS) T | PO4-CAGATCAGATCGGAAGAGCGAGCACATCCCTTTCTCACA |
| TCAAGT | CAAGCAGAAGACGGCATACGAGATCGGTCTCGGCATTCCTGCTGAACCGCTCTTCCGATCTTCAAGT (PS) T | PO4-ACTTGAAGATCGGAAGAGCGAGCACATCCCTTTCTCACA |
| CTGATC | CAAGCAGAAGACGGCATACGAGATCGGTCTCGGCATTCCTGCTGAACCGCTCTTCCGATCTCTGATC (PS) T | PO4-GATCAGAGATCGGAAGAGCGAGCACATCCCTTTCTCACA |
| AAGCTA | CAAGCAGAAGACGGCATACGAGATCGGTCTCGGCATTCCTGCTGAACCGCTCTTCCGATCTAAGCTA (PS) T | PO4-TAGCTTAGATCGGAAGAGCGAGCACATCCCTTTCTCACA |
| GTAGCC | CAAGCAGAAGACGGCATACGAGATCGGTCTCGGCATTCCTGCTGAACCGCTCTTCCGATCTGTAGCC (PS) T | PO4-GGCTACAGATCGGAAGAGCGAGCACATCCCTTTCTCACA |
| TACAAG | CAAGCAGAAGACGGCATACGAGATCGGTCTCGGCATTCCTGCTGAACCGCTCTTCCGATCTTACAAG (PS) T | PO4-CTTGTAAGATCGGAAGAGCGAGCACATCCCTTTCTCACA |
| CGTGAT | CAAGCAGAAGACGGCATACGAGATCGGTCTCGGCATTCCTGCTGAACCGCTCTTCCGATCTCGTGAT (PS) T | PO4-ATCACGAGATCGGAAGAGCGAGCACATCCCTTTCTCACA |
| AGAAGC | CAAGCAGAAGACGGCATACGAGATCGGTCTCGGCATTCCTGCTGAACCGCTCTTCCGATCTAGAAGC (PS) T | PO4-GCTTCTAGATCGGAAGAGCGAGCACATCCCTTTCTCACA |
| GTTCCG | CAAGCAGAAGACGGCATACGAGATCGGTCTCGGCATTCCTGCTGAACCGCTCTTCCGATCTGTTCCG (PS) T | PO4-CGGAACAGATCGGAAGAGCGAGCACATCCCTTTCTCACA |

* Phosphorothioate (PS) bond between the last two bases of all the PE 2 Adapter oligos. ^#^ All Walking Adapter 2 oligos were 5’ phosphorylated.

The first 6 barcode tags were taken from the Multiplexing DNA oligonucleotide sequences provided by Illumina. Barcodes in the adapter sequences are marked in red blocks.

The 5 nt barcode sequences incorporated with nested primers

were the barcodes that sequenced first in Read 1.
