# Supplementary material for: Recurrent Targeted Genes of Hepatitis B Virus in the Liver Cancer Genomes Identified by a Next-Generation Sequencing–Based Approach
Source: PLoS Genet. 2012 Dec 6;8(12):e1003065. doi: 10.1371/journal.pgen.1003065 (PMC3516541; doi:10.1371/journal.pgen.1003065)
Supplement: Table S3 — The 5 nt barcode sequences incorporated with nested primers. (DOC) [file pgen.1003065.s005.doc]

**Table S3:** The 5 nt barcode sequences incorporated with nested primers

| **5 nt Barcode Tags** | **PE1-Barcode-HBX2 Sequences** |
| --- | --- |
| AACCG | CACTCTTTCCCTACACGACGCTCTTCCGATCTaaccgACTTCGCTTCACCTCTGCACGT |
| AACGA | CACTCTTTCCCTACACGACGCTCTTCCGATCTaacgaACTTCGCTTCACCTCTGCACGT |
| AACTC | CACTCTTTCCCTACACGACGCTCTTCCGATCTaactcACTTCGCTTCACCTCTGCACGT |
| AAGCT | CACTCTTTCCCTACACGACGCTCTTCCGATCTaagctACTTCGCTTCACCTCTGCACGT |
| CCAAT | CACTCTTTCCCTACACGACGCTCTTCCGATCTccaatACTTCGCTTCACCTCTGCACGT |
| CTCTG | CACTCTTTCCCTACACGACGCTCTTCCGATCTctctgACTTCGCTTCACCTCTGCACGT |
| CTGGA | CACTCTTTCCCTACACGACGCTCTTCCGATCTctggaACTTCGCTTCACCTCTGCACGT |
| CTTCG | CACTCTTTCCCTACACGACGCTCTTCCGATCTcttcgACTTCGCTTCACCTCTGCACGT |
| GCGTA | CACTCTTTCCCTACACGACGCTCTTCCGATCTgcgtaACTTCGCTTCACCTCTGCACGT |
| GGACT | CACTCTTTCCCTACACGACGCTCTTCCGATCTggactACTTCGCTTCACCTCTGCACGT |
| GGTAA | CACTCTTTCCCTACACGACGCTCTTCCGATCTggtaaACTTCGCTTCACCTCTGCACGT |
| TCGTC | CACTCTTTCCCTACACGACGCTCTTCCGATCTtcgtcACTTCGCTTCACCTCTGCACGT |
| TCTGC | CACTCTTTCCCTACACGACGCTCTTCCGATCTtctgcACTTCGCTTCACCTCTGCACGT |
| TGAGC | CACTCTTTCCCTACACGACGCTCTTCCGATCTtgagcACTTCGCTTCACCTCTGCACGT |

Index sequences were designed following instructions on <http://bioinf.eva.mpg.de/multiplex/>. The lowercased in the middle of PE1-Barcode-HBX2 sequences were the barcodes that sequenced first in Read 1.
