# Supplementary material for: Recurrent Targeted Genes of Hepatitis B Virus in the Liver Cancer Genomes Identified by a Next-Generation Sequencing–Based Approach
Source: PLoS Genet. 2012 Dec 6;8(12):e1003065. doi: 10.1371/journal.pgen.1003065 (PMC3516541; doi:10.1371/journal.pgen.1003065)
Supplement: Table S10 — Clinical information of the tissues used in the study. (DOC) [file pgen.1003065.s012.doc]

**Table S9:** Clinical information of the tissues used in the study.

| **Admission number** | **Tissues** | | **Sex** | **Age** | **Serological markers** | | | |
| --- | --- | --- | --- | --- | --- | --- | --- | --- |
|  | **Cancer** | **Adjacent** |  |  | **HbsAg** | **HbeAg** | **anti-Hbe** | **anti-Hbc** |
| 481992 | C7 | N7 | M | 45 | + | - | - | - |
| 482367 | C9 | N9 | M | 53 | + | - | - | + |
| 483943 | C12 | N12 | M | 51 | + | + | - | - |
| 484509 | C13 | N13 | M | 60 | + | - | + | + |
| 485325 | C16 | N16 | M | 49 | + | - | - | - |
| 485124 | C17 | N17 | M | 63 | + | - | + | + |
| 483217 | C18 | N18 | M | 69 | + | + | + | - |
| 485282 | C19 | N19 | M | 36 | + | + | - | + |
| 486316 | C21 | N21 | F | 70 | + | - | + | + |
| 485897 | C22 | N22 | M | 67 | + | - | + | + |
| 486243 | C23 | N23 | M | 63 | + | - | + | + |
| 485994 | C24 | N24 | F | 59 | + | - | + | + |
| 486938 | C25 | N25 | M | 51 | + | - | + | + |
| 487363 | C28 | N28 | M | 64 | - | - | + | + |
| 494564 | C100 | N100 | F | 53 | + | + | + | + |
| 497590 | C101 | N101 | M | 39 | + | - | + | + |
| 496016 | C102 | N102 | M | 46 | + | + | - | + |
| 498087 | C103 | N103 | M | 48 | + | - | - | + |
| 502911 | C104 | N104 | M | 34 | + | + | + | - |
| 500076 | C105 | N105 | F | 46 | + | + | + | - |
| 493756 | C106 | N106 | F | 24 | + | - | + | + |
| 499663 | C107 | N107 | M | 46 | + | - | + | + |
| 499889 | C108 | N108 | M | 53 | - | - | - | - |
| 499038 | C109 | N109 | M | 75 | - | - | + | + |
